# TSPO contributes to neuropathology and cognitive deficits in Alzheimer’s disease

**DOI:** 10.1186/s12974-025-03583-4

**Published:** 2025-10-28

**Authors:** Kelly Ceyzériat, Aurélien M. Badina, Laurene Abjean, Léa Meyer, Farha Bouteldja, Marta Balkota, Quentin Amossé, Oriane Prudhomme, Ryan J. Middleton, Guo-Jun Liu, Richard B. Banati, Thomas Zilli, Aurelien Lathuiliere, David Owen, Pierre Maechler, Valentina Garibotto, Stergios Tsartsalis, Philippe Millet, Benjamin B. Tournier

**Affiliations:** 1 CIBM Center for Biomedical Imaging, Geneva, Switzerland; 2 Department of Psychiatry, University of Geneva, Geneva, Switzerland; 3 Department of Psychiatry, University Hospitals of Geneva, Avenue de La Roseraie, 64, Geneva, 1205 Switzerland; 4 Department of Cell Physiology and Metabolism, University of Geneva, Geneva, Switzerland; 5 Australian Nuclear Science and Technology Organisation (ANSTO), Sydney, Australia; 6 Brain and Mind Centre, Medical Imaging Science, Faculty of Medicine and Health, The University of Sydney, Sydney, Australia; 7 Santuario Accademico S. Giovanni D’Andorno, Casa Alpina ‘Principessa Laetitia’, Frazione Bele, Campiglia Cervo, 13812 Italy; 8 Faculty of Biomedical Sciences, Università Della Svizzera Italiana, Lugano, Switzerland; 9 Faculty of Medicine, University of Geneva, Geneva, Switzerland; 10 Department of Radiation Oncology, Oncology Institute of Southern Switzerland, EOC, Bellinzona, Switzerland; 11 Department of Rehabilitation and Geriatrics, Memory Center, Geneva University Hospital and University of Geneva, Geneva, Switzerland; 12 Department of Brain Sciences, Imperial College London, London, UK; 13 UK Dementia Research Institute at Imperial College London, London, UK; 14 Diagnostic Department, Division of Nuclear Medicine, University Hospitals and University of Geneva, Geneva, Switzerland

**Keywords:** TSPO, 3 × TgAD, Alzheimer’s disease, Astrocytes, Hippocampus

## Abstract

The 18kDa translocator protein (TSPO) is increased in neurodegenerative diseases. In Alzheimer’s disease (AD) animal models, TSPO’s upregulation is detected first in astrocytes, then in microglia. However, the role of TSPO in the pathophysiology and symptoms characteristic of the disease remains unknown. In the human *postmortem* hippocampus, we show here that TSPO correlated positively with reactive astrocyte-associated genes and negatively with genes involved in glycolytic pathways. In addition, we observed that TSPO deletion in 3 × TgAD mice reversed the reduction in glucose uptake and reduced astrocyte reactivity. We observed a decrease of poorly and highly aggregated forms of Tau (-44% and -82%, respectively) and Aβ42 (-25% and -95%, respectively) at 9 months of age. In 5 × FAD mice, we confirmed the association between TSPO, astrocyte reactivity and Aβ. Functionally, Tau over-expression in the hippocampus induced a memory decline in WT animals, prevented in TSPO^−/−^ mice. Altogether, these data demonstrate that TSPO plays an important role in the active progression of AD. This identifies TSPO as a potential therapeutic target and highlights the importance of astrocyte metabolism in the pathogenesis of the disease.

**Supplementary Information:**

The online version contains supplementary material available at 10.1186/s12974-025-03583-4.

## Introduction

Alzheimer’s disease (AD) is the most common form of dementia and represents a significant economic and social cost. It is characterized by a gradual accumulation of protein aggregates, including amyloid-β (Aβ) and Tau, and a progressive loss of cognitive functions in addition to brain atrophy [1]. The intraneuronal presence of both Aβ and Tau is well described and may be the basis of synaptic dysfunction and subsequent neuron death [1–5]. Beyond the role of neurons in AD, the relative importance of glial cells is increasingly acknowledged. Astrocyte reactivity and microglial activation involve changes in their morphology and gene expression signature [6–8]. These functional changes can be either protective or aggravating of the pathology depending on the area of the brain considered, the pathological stage and severity and the duration of their activation [9, 10]. A marker of their proliferation or activation state during acute but also chronic neurodegenerative disease, is the de novo expression of the 18kDa translocator protein (TSPO) [11, 12], an outer mitochondrial membrane protein [13]. TSPO in the brain is mainly expressed in non-neuronal cell types and its expression increases in glial cells in response to various stimuli, such as the presence of proinflammatory factors or Aβ [14–20]. Significant TSPO expression in glial cells is regularly seen at earlier stages of neurodegeneration and appears to correlate with the accumulation of Aβ plaques in the hippocampus of both human and rodent models [7, 11]. In addition, astrocytes become reactive earlier than microglia [16, 17, 21] and consistent with this notion, the increase in TSPO density due to astrocytes occurs prior to that observed in microglial cells in AD models [19]. Previous studies suggest that the absence of TSPO may reduce the reactivity of glial cells and inflammatory responses [22–24]. The TSPO ligand FEPPA was shown to reduce the lipopolysaccharide (LPS)-induced reactivity of astrocytes [25] supporting this hypothesis. Some studies evaluated TSPO-targeted strategies and reported improvement of both inflammation and AD markers [26–30]. However, the possibility of off-target effects cannot be definitively excluded [31, 32]. In the human brain, microglial TSPO does not appear to reflect increased microglial activity or disease progression, as TSPO density in microglia remains unchanged both near Aβ plaques and in AD compared to controls [12, 14, 19]. In contrast to microglial TSPO, astrocytic TSPO remains largely understudied. The aim of this study was therefore to investigate the role of TSPO, with a particular focus on astrocytic TSPO in AD.

However, beyond its role in regulating glial cell reactivity, TSPO has also been shown to influence astrocyte metabolism in cell culture [33]. Astrocytes consume approximately 85% of the glucose in the brain, most of which is used for lactate synthesis via the conversion of pyruvate to lactate [34–36]. In addition, mitochondrial pyruvate catabolism is mainly mediated by pyruvate carboxylase, which leads to gluconeogenesis and eventually lactate production [37]. According to the astrocyte-neuron lactate shuttle hypothesis, astrocytes supply neurons with metabolic support by releasing lactate as an energy substrate [38, 39]. Astrocytic lactate has been shown to play a fundamental role in memory mechanisms since blocking the astrocyte-neuron lactate transfer inhibits memory formation and synaptic plasticity [40, 41]. Lactate has also been shown to exhibit neuroprotective properties [42] and high brain lactate levels are associated with overall better cognitive performances [43]. Thus, the early reduction in glucose consumption by astrocytes in AD [35] could play a major role in the etiology and evolution of AD.

In this context, we hypothesize that TSPO regulates astrocyte reactivity and metabolism early in the disease, impacts the development of the pathology and the accumulation of protein markers. In this study, we investigated TSPO expression in the human hippocampus and its association with AD molecular signatures. In contrast to humans, where it is not possible to predict the onset of pathology, mouse models allows the investigation of physiological changes at very early stages. It has been shown that the neuroinflammatory response, as well as TSPO accumulation, occurs prior to the appearance of Aβ plaques and intraneuronal Tau [44–47]. Then, to gain insight into the early contribution of TSPO to AD pathology, we analyzed its involvement in disease progression in the 3xTgAD mouse model at pre-plaque stages. Using global TSPO knockout mice (Tspo^tm1GuMu(GuwiyangWurra)^) [48], avoiding off-target effects, we measured the emergence and evolution of pathological markers. We studied 2-month-old animals to assess the impact on brain development, 4-month-old to simulate young adults and 9-month-old to mimic a very early stage of the pathology, when Aβ deposits are not yet observed in the hippocampus. At 9 and 16 months of age, glucose uptake was measured by [^18^F]Fluorodeoxyglucose ([^18^F]FDG) PET imaging and at the latter age, mRNA sequencing was applied to study the involvement of different cell types in the pathology. Finally, the impact of overexpression of Tau in the hippocampus on spatial working memory was assessed in wild type (WT) and TSPO^−/−^ mice.

## Results

### TSPO is associated with glycolysis and astrocyte markers in the human AD hippocampus

**Fig. 1:**
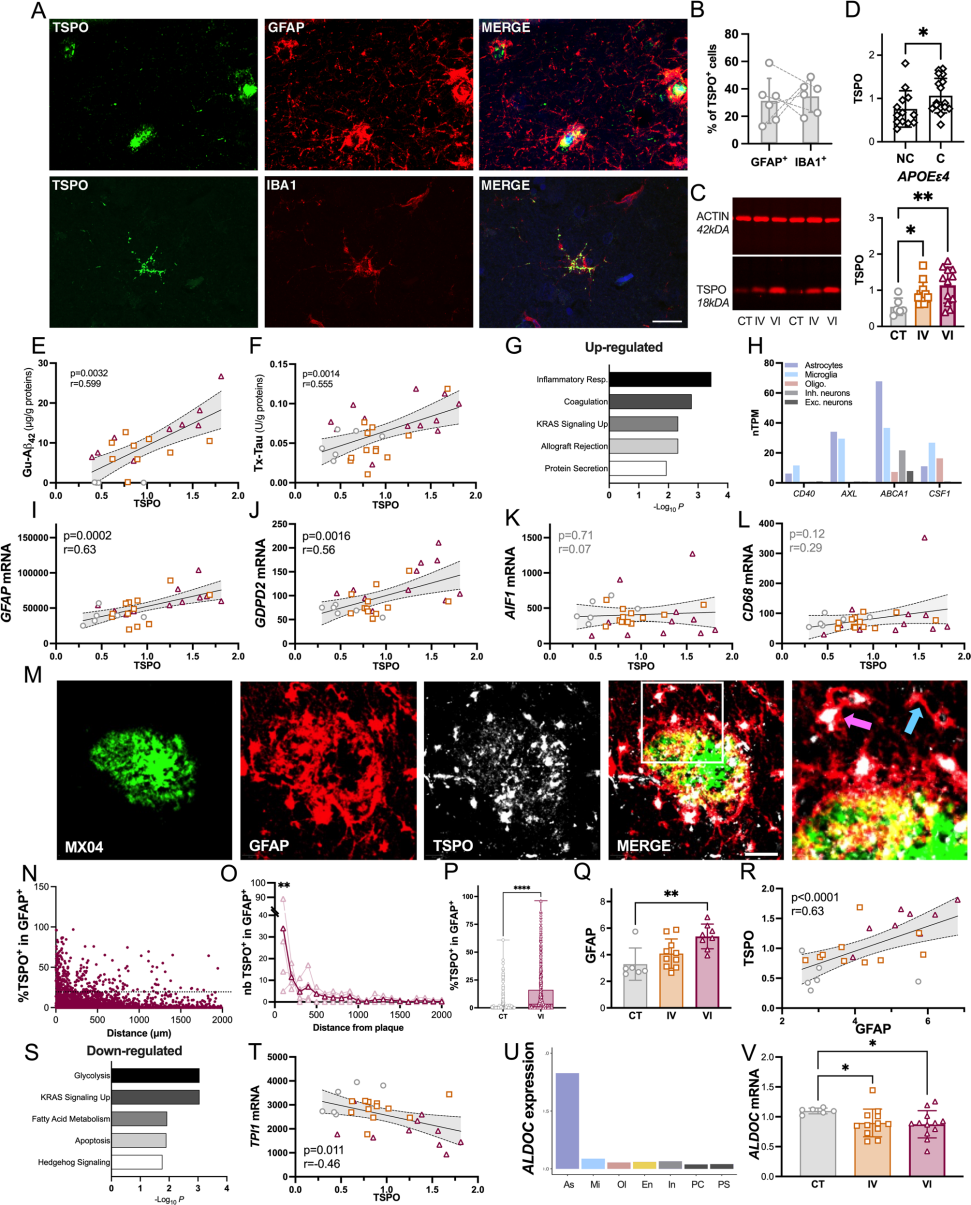
TSPO increase is associated with glycolysis and astrocyte markers in the human AD hippocampus. **A** TSPO (green) is associated with astrocytes (GFAP, red, top panel) and microglia (IBA1, red, bottom panel) in the human hippocampus, as shown in the representative control subject (female, 76-year-old, PMD: 51 h). Scale bar: 20 µm. **B** Confocal analysis of the fraction of GFAP^+^ and IBA1^+^ cells expressing TSPO (%) in the human hippocampus. **C** TSPO (TSPO/ACTIN ratio) in the hippocampus expressed as function of control (CT, n = 6), Braak IV (IV, n = 12) and Braak VI (VI, *n* = 13) subjects. Data are presented as individual values and mean ± SD and analyzed by the Kruskal–Wallis test with the uncorrected Dunn’s post hoc test (*H3* = 6.97, *P* = 0.03) **D** TSPO (TSPO/ACTIN ratio) expressed as function of ApoEε4 genotype (non-ApoEε4 carrier: NC, and ApoEε4 carrier: C). Data are presented as individual values and mean ± SD (*n* = 30) and analyzed by the Mann–Whitney test (*U* = 61, *P* = 0.039). **E–F** Correlation analysis (Pearson’s coefficient) between TSPO (TSPO/ACTIN ratio) and Guanidine-soluble Aβ_42_ (Gu-Aβ_42_, µg/g proteins, ELISA test) and Triton-soluble Tau (Tx-Tau, U/g proteins, ELISA test). **G** Functional enrichment analysis using enrichR with the human molecular signatures database applied on genes whose expression is positively correlated with TSPO levels. **H** Normalized transcripts per million (nTPM) of genes involved in the inflammatory response (as defined in G) from the human protein atlas database. **I-L** Correlation analysis (Pearson's coefficient) between TSPO (TSPO/ACTIN ratio) and mRNA expression levels of specific astrocytes (I,J) or microglial (K,L) genes. **M** Representative example of the GFAP (red) fraction covered by TSPO (white) around a Aβ plaque (MXO4, green). Pink and blue stars in the insert show astrocyte with high and low TSPO levels, respectively. Scale bar: 40 µm. **N** Expression of TSPO in astrocytes (%TSPO.^+^) as a function of distance from the Aβ plaques. Each dot represents an astrocyte. The horizontal bar shows the threshold at 20%. **O** Quantification of the number of astrocytes with a TSPO staining > 20%, as a function of distance from the Aβ plaques. Pink and violet plots represent individual values and the average, respectively. **P** Expression of TSPO in astrocytes in CT and Braak VI subjects. Data are presented as individual values and min/max box (number of astrocytes on 100 µm in 4 CT: 290, number of astrocytes on 100 µm from an Aβ deposit in 4 Braak VI: 608) and analyzed by the Mann–Whitney test (*U* = 47,884, *P* < 0.0001). **Q** GFAP (GFAP/ACTIN ratio) in the hippocampus of CT (*n* = 6), Braak IV (*n* = 10) and Braak VI (*n* = 8) subjects. Data are presented as individual values and mean ± SD and analyzed by the Kruskal–Wallis test with the uncorrected Dunn's post hoc test (*P* < 0.05). **R** Correlation analysis (Pearson's coefficient) between GFAP/ACTIN and TSPO/ACTIN ratios. **S** Functional enrichment analysis using enrichR with the human molecular signatures database applied on genes whose expression is negatively correlated with TSPO. **T** Correlation analysis (Pearson’s coefficient) between TSPO (TSPO/ACTIN ratio) and mRNA expression levels of the *TPI1* gene expression. **U** Cell origin of *ALDOC* gene expression as measured by the expression weighted cell type enrichment method. As: astrocyte, En: endothelial cell, In: interneurons, Mi: microglia, PC: pyramidal CA1 neurons, PS: pyramidal SS neurons. **V** *ALDOC* mRNA levels in the hippocampus of CT (*n* = 6), Braak IV (*n* = 12) and Braak VI (*n* = 12) subjects. Data are presented as individual values and mean ± SD and analyzed by the Kruskal–Wallis test with the uncorrected Dunn's post hoc test (*H3* = 7.03, *P* = 0.029)

Figure 1A and B shows that, in the human AD hippocampus, TSPO colocalized with GFAP^+^ and IBA1^+^ staining, demonstrating its expression by astrocytes and microglia, respectively. We showed that TSPO density was increased in AD: hippocampus samples from Braak IV and Braak VI subjects showed significantly higher protein levels than controls (Fig. 1C). In addition, TSPO density was increased in APOEe4 carriers (*P* = 0.039, Fig. 1D). The accumulation of TSPO positively correlated with that of amyloid-β (Gu-Aβ42: *r* = 0.599, *P* = 0.0032, Fig. 1E; Gu-Aβ40: *r* = 0.636, *P* = 0.0012, supp. Fig. 1) and Tau (Tx-Tau: *r* = 0.555, *P* = 0.0014, Fig. 1F), suggesting that TSPO might be involved in the mechanisms of the disease progression. No correlation was found between TSPO and soluble forms of Aβ or aggregated forms of Tau (Tx-Aβ42: *r* = 0.215, *P* = 0.253; Tx-Aβ40: *r* = 0.088, *P* = 0.641; Gu-Tau: *r* = 0.092, *P* = 0.626, supp. Fig. 1).

To determine the changes linked to TSPO upregulation, we analyzed the expression of 770 genes related to inflammation. It revealed that TSPO expression was positively correlated with the expression of 36 genes and negatively correlated with the expression of 110 genes. Pathway enrichment analysis of the 36 positively correlated genes with the Human Molecular Signatures Database (MSigDB) revealed that the inflammatory response was the main upregulated pathway (Fig. 1G). This pathway is composed of 4 genes (*CD40*, *AXL*, *ABCA1* and *CSF1*) who are mainly expressed by astrocytes and microglia, as revealed by expression weighted cell type enrichment analysis (EWCE, Fig. 1H). To delve deeper into the links between TSPO and glial cells, we observed a positive correlation between TSPO and the astrocyte mRNA markers *GFAP* (r = 0.63, *P* = 0.0002), *GDPD2* (*r* = 0.56, *P* = 0.0016) and *SOX9* (*r* = 0.46, *P* = 0.0104), suggesting a direct link between TSPO and astrocyte reactivity (Fig. 1J, K and supp. Fig. 1). Conversely, we observed no association between TSPO density and the microglial *AIF1* (*r* = 0.07, *P* = 0.71) and *CD68* (*r* = 0.29, *P* = 0.12) mRNA markers (Fig. 1K, L).

We measured the relationship between astrocytic TSPO and Aβ deposits through proximity analysis. An increase in astrocytic TSPO density was observed both in proximity to Aβ plaques (*P* < 0.01, Fig. 1M-O) and globally in Braak VI subjects compared to controls (*P* < 0.001, Fig. 1P). In contrast, no such increase was reported in microglial TSPO density [12]. Interestingly, the increase in GFAP density (*P* < 0.01, Fig. 1Q) was proportional to that of TSPO over the course of the disease (*r* = 0.63, *P* = 0.0009, Fig. 1R). In contrast to the increase in GFAP(at both mRNA and protein levels), we observed a reduction in the expression of *AMIGO2* and *S100A* gene expression as the disease progresses, but with no direct link to TSPO density (supp. Fig. 1).

On the 110 negatively correlated genes, pathway enrichment analysis revealed that glycolysis was the main altered pathway (Fig. 1S). We measured the expression of enzymes involved in glycolysis and TCA cycle activation, and 6 of them decreased and negatively correlated with the expression of TSPO: *TPI1* (*r* = −0.46, *P* = 0.011), *ENO2* (*r* = −0.52, *P* = 0.003), *PGK1* (*r* = −0.39, *P* = 0.032), *PGAM1*(*r* = −0.40, *P* = 0.029), *PDHA1* (*r* = −043, *P* = 0.018), *GOT1* (*r* = −0.41, *P* = 0.026) (Fig. 1T and supp. Fig. 1).

Among the enzymes involved in glycolysis, *ALDOC* is essentially expressed by astrocytes (EWCE analysis, Fig. 1U) and its expression was reduced as early as the Braak IV stage (Braak IV vs CT: *P* = 0.0125; Braak VI vs CT: *P* = 0.0199, Fig. 1V) that may suggest that astrocytes play at least in part a role in the reduction of glycolysis in AD, as previously suggested [49, 50]

### TSPO knockout stimulates glycolysis pathways in the hippocampus of 3 × TgAD mice

**Fig. 2:**
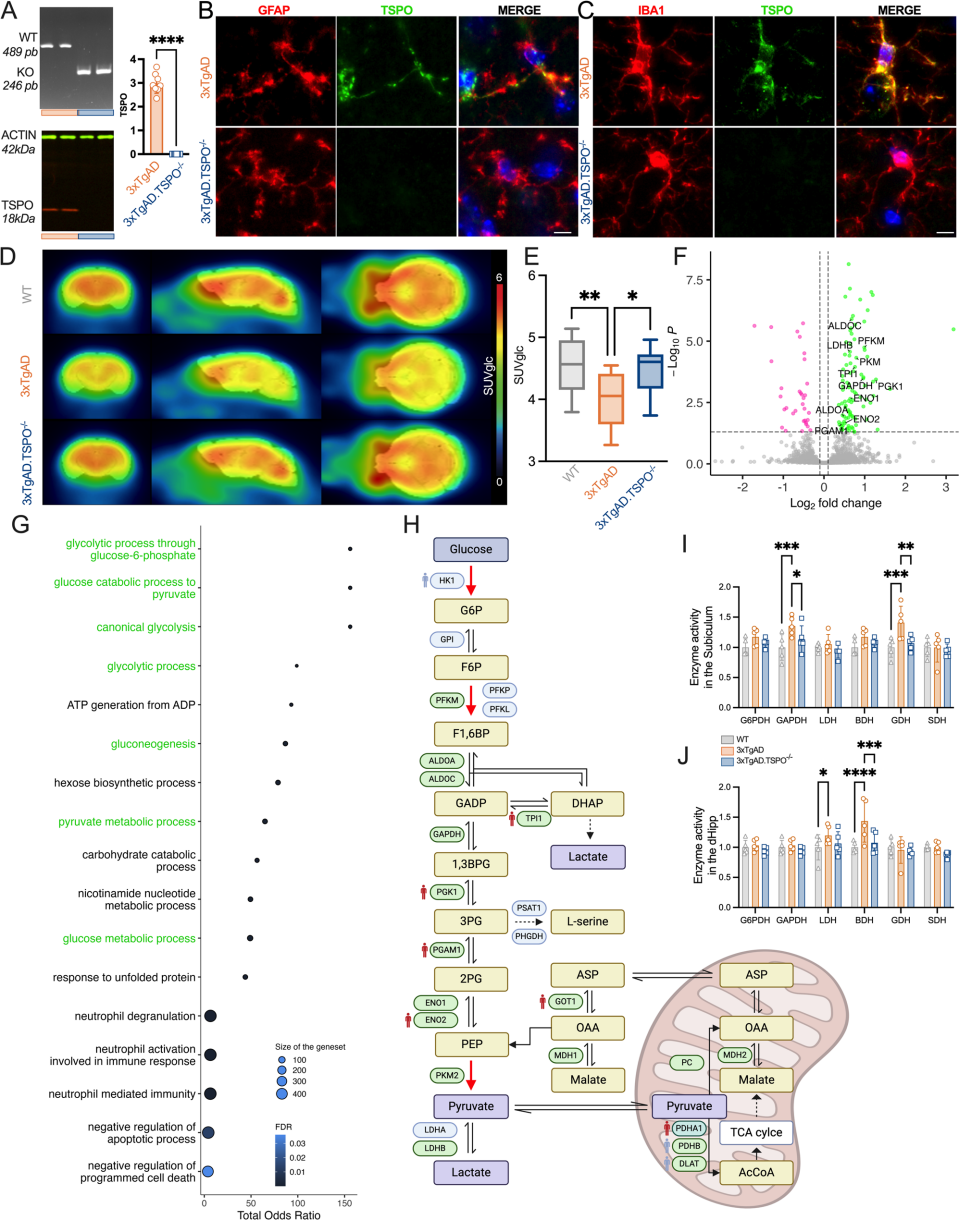
TSPO knock-out stimulates glycolysis pathways in the hippocampus of 3xTgAD mice. **A** Validation of the TSPO knock-out. Top: gDNA PCR gel shows the discrimination of size of *Tspo* amplicon, validating the knock-out in 3xTgAD.TSPO^−/−^ mice. Bottom and right: TSPO protein quantification by western blot in the hippocampus confirmed the abolition of TSPO in 3xTgAD.TSPO^−/−^ mice (two-tailed unpaired t-test: *P* < 0.0001). **B-C** Representative images of TSPO (green) staining in astrocytes (GFAP^+^, red, B) or in microglia (IBA1^+^, red, C). Scale bar: 20µm. **D-E** Mean parametric images and quantification of [^18^F]FDG uptake (SUVglc) in WT, 3xTgAD and 3xTgAD.TSPO^−/−^ mice (*n* = 10/group; two-way ANOVA, genotype,* F2,513* = 4.89, *P* = 0.0078; brain area, *F18,513* = 1.64, *P* = 0.049; genotype x brain area, *F*3*6,513* = 0.029, *P* > 0.05). **F** Volcano plot of the differentially expressed genes in the hippocampus of 3xTgAD.TSPO^−/−^ as compared to 3xTgAD mice. Dots represent individual gene whose the expression is downregulated (purple), upregulated (green) or unaltered (grey, |log_2_FC|≥ 0.1 or adjusted *P* value > 0.05) in 3xTgAD.TSPO^−/−^ mice. **G** Functional enrichment analysis using R studio with the GO biological process database. Glycolysis pathways are indicated in green. **H** Schematic representation of products and enzymes involved in glycolysis pathways. Red arrows indicate limiting steps. Enzymes are highlighted in gray or green, depending on whether their density is unchanged or increased in 3xTgAD.TSPO^−/−^ mice, respectively. The PDHA1 density tended to increase. For comparison, enzymes whose expression levels are or are not correlated with TSPO in the human hippocampus are indicated by red and blue human, respectively. This representation was made with Biorender. **I-J** Metabolic mapping in the subiculum and the dorsal hippocampus (dHipp) of WT, 3xTgAD and 3xTgAD.TSPO^−/−^ mice. Data are presented as individual values and mean ± SD (*n* = 5/group) and analyzed by the Two-way ANOVA test with a LSD post hoc test (I: genotype,* F2,72* = 12.2, *P* < 0.0001; enzyme, *F5,72* = 3.49, *P* = 0.007; genotype x enzyme, *F10,72* = 1.41, *P* > 0.05; J: genotype,* F2,72* = 6.92, *P* = 0.0018; enzyme, *F5,72* = 4.67, *P* = 0.0009; genotype x enzyme, *F10,72* = 1.89, *P* = 0.059). Abbreviations in H: 1,3BPG: 1,3-Bisphosphoglycerate; 2PG: 2-Phosphoglycerate; 3PG: 3-Phosphoglycerate; AcCoa: Acetyl-CoA; ALDOAC: Aldolase A/C; ASP: Aspartate; DHAP: Dihydroxyacetone phosphate; DLAT: Dihydrolipoyl transacetylase; ENO1/2: Enolase ½; F1,6BP: Fructose-1, 6-phosphate; F6P: Fructose-6-phosphate; GAPD: Glyceraldehyde 3-phosphate; GAPDH: Glyceraldéhyde-3-phosphate dehydrogenase; G6P: Glucose-6-phosphate; GOT1: Aspartate aminotransferase; GPI: Glucose-6-phosphate isomerase; HK1: Hexokinase 1; LDHA/B: Lactate dehydrogenase A/B; MDH1/2: Malate dehydrogenase 1/2; OAA: Oxaloacetic acid; PC: Pyruvate carboxylase; PDHA1/B: Pyruvate dehydrogenase E1 subunit alpha 1/beta; PEP: Phosphoenolpyruvate; PFKM/P/L: Phosphofructokinase muscle/platelet/liver type; PGAM1: Phosphoglycerate mutase; PGK1: Phosphoglycerate kinase; PKM2: Pyruvate kinase muscle isoform 2; TPI1: Triose-phosphate isomerase

Having shown a negative correlation between TSPO protein expression and expression of genes related to glycolysis, we then tested whether TSPO has a causal role in glycolysis repression, elevation in astrocytic reactivity, Aβ and Tau accumulation in AD. We knocked out the gene encoding the TSPO protein in an AD mouse model by crossing TSPO^−/−^ (Tspo^tm1GuMu(GuwiyangWurra)^) with 3xTgAD mice, thereby creating 3xTgAD.TSPO^−/−^ mice. We confirmed the absence of DNA and protein TSPO levels using gDNA PCR and western blotting, respectively (Fig. 2A). Immunofluorescence confirmed the loss of TSPO colocalization with GFAP ad IBA1 in 3xTgAD.TSPO^−/−^ mice (Fig. 2B, C).

Glucose uptake was first estimated using PET imaging with [^18^F]FDG in 9-month-old WT, 3xTgAD and 3xTgAD.TSPO^−/−^ mice. Figure 2D shows parametric images of the mean [^18^F]FDG accumulation in all groups (SUVglc) and the quantification revealed a whole brain hypometabolism in 3xTgAD (*P* < 0.01) reversed by the knock-out of TSPO (*P* < 0.05, Fig. 2E). To determine which proteins were involved in this mechanism, we performed a LC–MS/MS-based quantitative proteomic analysis. We quantified 2581 proteins, in the range of previous studies in mice [51–53]. Among those, 128 proteins were significantly deregulated, 95 were up-regulated and 33 were down-regulated in 3xTgAD.TSPO^−/−^ mice relative to 3xTg-AD, as shown in the volcano plot in Fig. 2F. Significantly altered pathways revealed proteins (Fig. 2F) and pathways (Fig. 2G) involved in glucose metabolism. A schematic representation of glycolysis pathways shows that of the 24 enzymes measured, 17 had increased density and 1 tended to increase in 3xTgAD.TSPO^−/−^ mice (Fig. 2H). Interestingly, several of them were correlated to TSPO levels in humans (TPI1, PGK1, PGAM1, GOT1, ENO2, and PDHA1, see Fig. 1 and supp. Fig. 1 for details). Among these proteins, ALDOC -essentially expressed by astrocytes- was increased, suggesting an astrocyte-mediated effect on brain glucose metabolism in TSPO knockout.

To further investigate changes in metabolic pathways, we measured the activity of metabolic enzymes, directly in cryopreserved sub-brain regions, representing various metabolic pathways. In the subiculum, we observed an increased activity of GAPDH (glycolysis reporter, *P* = 0.0009) and GDH (amino acid metabolism reporter, *P* = 0.0001) in 3xTgAD mice compared to WT. This increased activity was normalized in 3xTgAD.TSPO^−/−^ mice (*P* = 0.037 and *P* = 0.0014, Fig. 2I). In the dorsal hippocampus, we observed an increased activity of LDH (anaerobic pathway reporter, *P* = 0.043) and BDH (ketolysis reporter, *P* < 0.0001) in 3xTgAD mice. The BDH increased activity was also normalized in 3xTgAD.TSPO^−/−^ mice (*P* = 0.0003, Fig. 2J). The activity of the enzymes representative of pentose phosphate pathway (G6PDH) and mitochondrial aerobic pathway (SDH) were unchanged. In the ventral hippocampus, a two-way ANOVA of enzymatic activity confirmed a genotype effect between 3xTgAD and WT (*P* = 0.006), and a normalization in the 3xTgAD.TSPO^−/−^ mice (3xTgAD vs 3xTgAD.TSPO^−/−^, *P* = 0.0005; WT vs 3xTgAD.TSPO^−/−^, *P* > 0.05, supp. Fig. 2).

### TSPO knockout reduces astrocyte reactivity in the hippocampus of 3 × TgAD mice

**Fig. 3:**
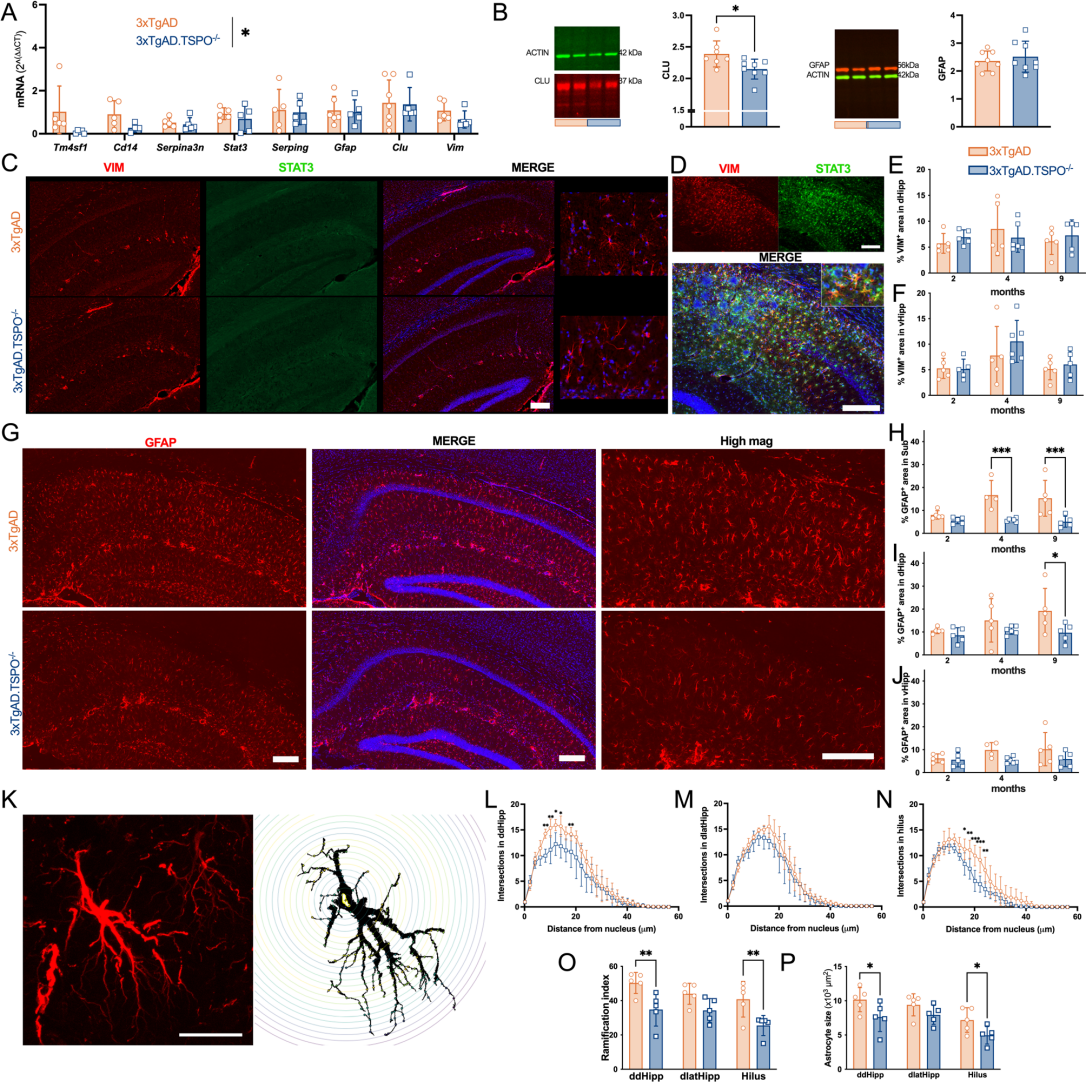
3xTgAD.TSPO^−/−^ mice exhibit reduced astrocyte reactivity. **A** Quantification of mRNA in the whole hippocampus (two-way ANOVA, genotype,* F1,66* = 4.71, *P* = 0.033; gene, *F7,66* = 2.37, *P* > 0.05; genotype x gene, *F7,66* = 0.58, *P* > 0.05). **B** Representatives immunoblot (showing two samples from 3xTgAD and two samples from 3xTgAD.TSPO^−/−^ mice) and protein quantification (normalized to ACTIN) for CLUSTERIN (CLU, two-tailed unpaired t-test: *P* = 0.024) and GFAP (two-tailed unpaired t-test: *P* > 0.05). **C** Representative example of VIMENTIN (VIM, red), STAT3 (green) immunoreactivity and DAPI (blue in merge images) in 9-month-old 3xTgAD and 3xTgAD.TSPO^−/−^ mice. Scale bar: 200 µm (inserts display high magnification). **D** Positive control for VIM (left), STAT3 (right) immunoreactivity and merge images with DAPI (20-month-old 3xTgAD mouse). Scale bar: 200 µm. **E–F** Quantification of % of positive VIM-ir area in dHipp (E) and vHipp (F, %VIM^+^, two-way ANOVA, dHipp: genotype x age, *F2,25* = 0.78, *P* > 0.05; genotype,* F1,25* = 0.04, *P* > 0.05; age, *F2,25* = 0.56, *P* > 0.05; vHipp: genotype x age, *F2,25* = 0.49, *P* > 0.05; genotype,* F1,25* = 0.92, *P* > 0.05; age, *F2,25* = 4.36, *P* = 0.024). **G** Representative example of DAPI (blue) staining and GFAP (red) immunoreactivity at two magnifications in 9-month-old 3xTgAD and 3xTgAD.TSPO^−/−^ mice. Scale bar: 200 µm. **H-J** Quantification of % of positive GFAP-ir area in the subiculum (Sub, H), dorsal hippocampus (dHipp, I) and ventral hippocampus (vHipp, J) (%GFAP^+^, two-way ANOVA, Subiculum: genotype x age, *F2,24* = 3.16, *P* = 0.06; genotype,* F1,24* = 26.35, *P* < 0.0001; age, *F2,24* = 3.03, *P* = 0.06; dHipp: genotype x age, *F2,25* = 1.06, *P* > 0.05; genotype,* F1,25* = 6.19, *P* = 0.019; age, *F2,25* = 1.75, *P* > 0.05; vHipp: genotype x age, *F2,24* = 0.83, *P* > 0.05; genotype,* F1,24* = 5.07, *P* = 0.034; age, *F2,24* = 0.86, *P* > 0.05). **K-N** Sholl analysis of astrocytes in 9-month-old 3xTgAD and 3xTgAD.TSPO.^−/−^ mice. Representative example of the signal extraction for Sholl analysis (K, GFAP, red). Scale bar: 20 µm. Quantification of the number of intersections as function of the distance from soma in dorso-dorsal (ddHipp, L), dorso-lateral (dlatHipp, M) hippocampus and hilus (N, wo-way ANOVA, ddHipp: genotype x distance, *F28,224* = 3.02, *P* < 0.0001; genotype,* F1,8* = 5.34, *P* = 0.049; distance, *F28,224* = 129.7, *P* < 0.0001; dlatHipp: genotype x distance, *F28,224* = 0.88, *P* = 0.63; genotype,* F1,8* = 1.1, *P* > 0.05; distance, *F28,224* = 132.9, *P* < 0.0001; hilus: genotype x distance, *F28,224* = 4.15, *P* < 0.0001; genotype,* F1,8* = 5.96, *P* = 0.04; distance, *F28,224* = 151.9, *P* < 0.0001). **O** Total number of ramifications (two-way ANOVA: genotype x area, *F2,24* = 0.46, *P* > 0.05; genotype,* F1,24* = 22.87, *P* < 0.001; area, *F2,24* = 3.80, *P* = 0.036). **P** Size of the soma of astrocytes (two-way ANOVA: genotype x area, *F2,24* = 0.26, *P* > 0.05; genotype,* F1,24* = 11.4, *P* = 0.0025; area, *F2,24* = 8.71, *P* = 0.0014)

To investigate how astrocyte reactivity is modulated by TSPO knockout, we analyzed multiple markers of reactive astrocytes at the mRNA, protein and morphological levels. The expression of genes involved in the reactivity of astrocytes (*Tm4sf1, Cd14, Serpina3n, Stat3, Serping, Gfap, Clu, Vim*) was globally reduced in 3xTgAD.TSPO^−/−^ mice compared to 3xTgAD mice (Fig. 3A), suggesting a decrease of their reactive state. CLUSTERIN, a protein secreted by astrocytes and known to promote formation of extracellular Aβ deposits [54, 55], and an AD GWAS gene, was significantly reduced in 3xTgAD.TSPO^−/−^ mice (−9±1.2%, *P* = 0.024, Fig. 3B), further suggesting a reduction in the pro-inflammatory state of astrocytes [56]. As previous studies reported that the reactivity of astrocytes, as well as the presence of AD markers (Aβ, Tau) and the expression of TSPO are heterogeneous depending on the location in the hippocampus [47, 57, 58], we conducted separate analyses of the subiculum, dorsal and ventral sub-divisions of the hippocampus. Because they are modulated in reactive astrocytes, we investigated VIMENTIN (VIM) and the canonical inducer of astrogliosis STAT3 (signal transducer and activator of transcription 3), both modulated in reactive astrocytes, using immunofluorescence. At 9 months of age, 3xTgAD and 3xTgAD.TSPO^−/−^ mice were negative for STAT3 in comparison with the clear positive staining in aged (20-month-old) 3xTgAD control animals (Fig. 3C-D). In addition, a dimly VIM staining was observed in 9-month-old 3xTgAD and 3xTgAD.TSPO^−/−^ mice in comparison with the positive old controls (Fig. 3C-D), confirming the very early stage of our study documented by the absence of STAT3 [59]. The VIM positive area did not differ between groups in the dorsal and the ventral hippocampus (Fig. 3E,F) and the VIM signal in the subiculum was below detection threshold. As VIM from endothelial cells may interfere with astrocytic VIM, we measured the degree of GFAP^+^ area in VIM^+^ area. We observed that most of the VIM^+^ signal was expressed by GFAP^+^ cells. More than 83% of the staining were colocalized with GFAP^+^ in the *stratum oriens, stratum radiatum* and *stratum lacunosum-moleculare* but only of 61.4% colocalized in the molecular layer of dentate gyrus, which may indicate an endothelial origin for the VIM^+^ signal (supp. Fig. 3). Then, we quantified VIM^+^ signal in these hippocampus subdivisions separately and confirmed that there is no difference between 9-month-old 3xTgAD and 3xTgAD.TSPO^−/−^ mice (supp. Fig. 3).

Finally, we also analyzed GFAP as one of the earliest and most representative markers of astrocyte reactivity. Over the whole hippocampus, the absence of TSPO did not alter GFAP density (Fig. 3B). Conversely, the analysis of hippocampus subdivisions revealed differences in GFAP density. Representative images of GFAP immunoreactivity in the dorsal part of the hippocampus clearly show a reduction in 3xTgAD.TSPO^−/−^ mice (Fig. 3G), confirmed by the quantification at 9-months-old (−50 ±7.5%, Fig. 3I). In the subiculum, the quantification revealed a reduction in the area occupied by astrocytes in 3xTgAD.TSPO^−/−^ mice at 4 and 9 months of age (−65 ±1.6% and −67 ±5.6%, respectively, Fig. 3H), further suggesting a decreased astrocyte reactivity. However, astrocytes in the ventral hippocampus were not affected by the absence of TSPO (Fig. 3J). To validate whether these effects were specific for 3xTgAD.TSPO^−/−^ mice, a comparative analysis was conducted in WT and TSPO^−/−^ mice. In this case, no difference between WT and TSPO^−/−^ was observed (supp. Fig. 4).

To further examine astrocyte changes, we analyzed their morphological complexity by measuring branching, defined as the number of branches relative to the distance from the soma (Fig. 3K). There was a reduction in 3xTgAD.TSPO^−/−^ mice at the level of the dorso-dorsal hippocampus and the hilus but not at the level of the dorso-lateral hippocampus (Fig. 3L-N). The total branching number and the size of astrocyte soma were also significantly reduced in the dorso-dorsal hippocampus and hilus of 3xTgAD.TSPO^−/−^ mice (Fig. 3O, P), confirming lower activation of astrocytes.

In contrast, TSPO knockout did not alter classical markers of microglial reactivity. Indeed, microglial and inflammatory gene expression was unchanged in 3xTgAD.TSPO^−/−^ mice compared to 3xTgAD at 9 months of age (supp. Fig. 5 A, B). Neither IBA^+^ area or microglial cell morphology was altered by TSPO deletion (supp. Fig. 5C-K), supporting the idea that the effect of TSPO knockout on astrocytes is not mediated through microglia. In addition, IBA^+^ area is not modified between WT and TSPO^−/−^ mice (supp. Fig. 4).

### TSPO knockout decreases Aβ and Tau neuropathology in the hippocampus of 3xTgAD mice

**Fig. 4:**
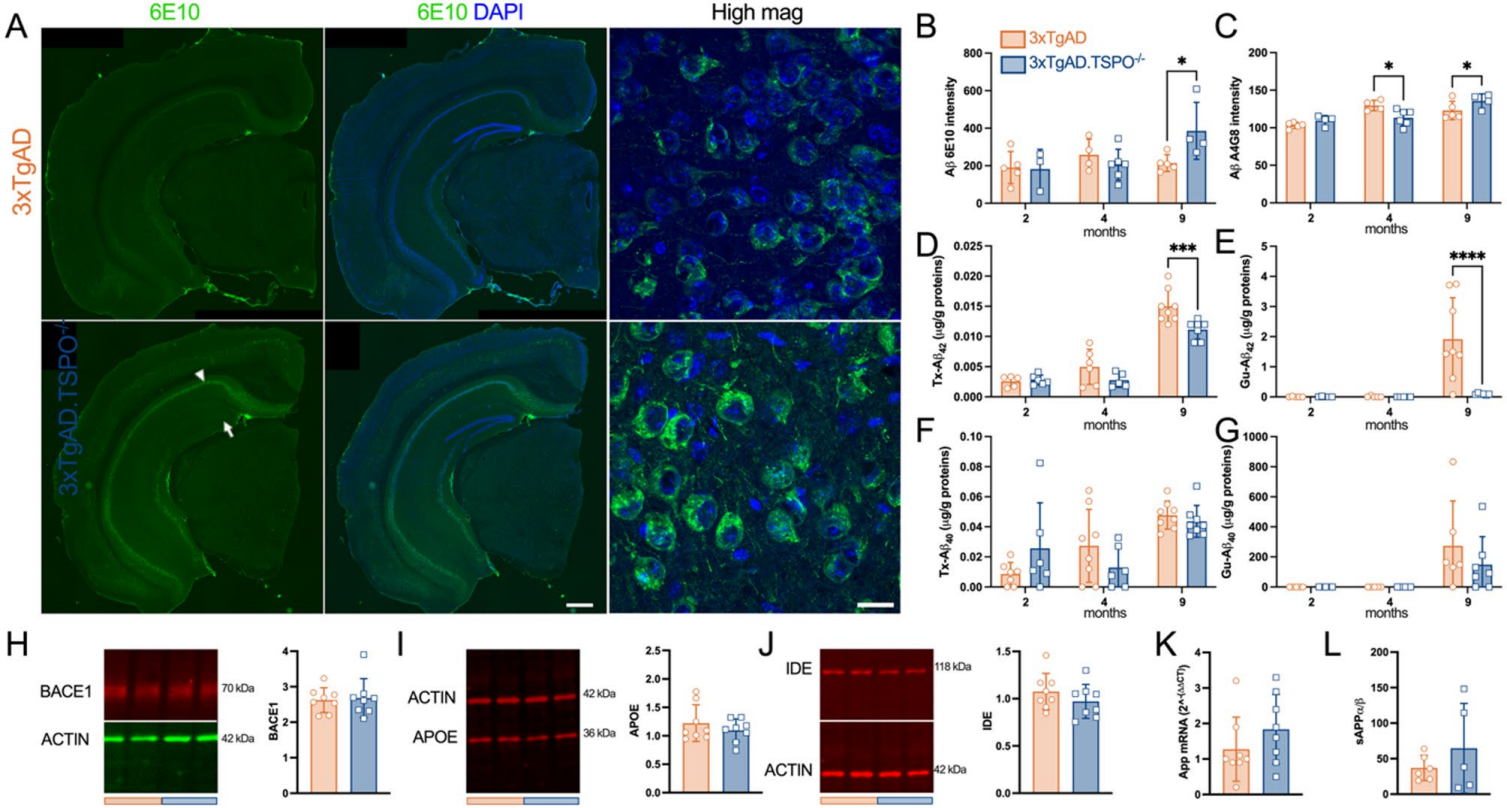
3xTgAD.TSPO^−/−^ mice exhibit reduced and delayed amyloid pathology. **A** Representative example of 6E10 perinuclear immunoreactivity (-ir, green) and DAPI (blue) in 9-month-old 3xTgAD and 3xTgAD.TSPO^−/−^ mice. Scale bar: 500 µm and 20 µm in high magnification (High mag) images. Arrow indicates the granule neurons of the hippocampal dentate gyrus that were negative for 6E10-ir and arrowhead indicates the pyramidal neurons that were positive for 6E10-ir. Note the absence of extracellular Aβ deposits. **B** Quantification of 6E10-ir perinuclear labeling (two-way ANOVA: genotype x age, *F2,22* = 6.59, *P* = 0.0057; genotype,* F1,22* = 0.07, *P* > 0.05; age, *F2,22* = 14.92, *P* < 0.0001). **C** Quantification of 4G8-ir intensity (two-way ANOVA: genotype x age, *F2,21* = 3.60, *P* = 0.045; genotype,* F1,21* = 1.00, *P* > 0.05; age, *F2,21* = 3.07, *P* = 0.067). **D-G** Quantification of triton × 100- (Tx) and guanidine- (Gu) soluble forms of Aβ42 (D-E) and Aβ40 (F-G) (two-way ANOVA, Tx-Aβ42: genotype x age, *F2,31* = 3.33, *P* = 0.049; genotype,* F1,31* = 8.66, *P* = 0.0061; age, *F2,31* = 109.5, *P* < 0.0001; Tx-Aβ40: genotype x age, *F2,37* = 2.79, *P* > 0.05; genotype,* F1,37* = 0.006, *P* > 0.05; age, *F2,37* = 11.91, *P* = 0.0001; Gu-Aβ42: genotype x age, *F2,28* = 7.13, *P* = 0.003; genotype,* F1,28* = 6.53, *P* = 0.016; age, *F2,28* = 8.63, *P* = 0.0012; Gu-Aβ40: genotype x age, *F2,28* = 0.67, *P* > 0.05; genotype,* F1,28* = 0.63, *P* > 0.05; age, *F2,28* = 7.64, *P* = 0.0023). **H-J** Quantification of protein in the whole hippocampus of 9-month-old mice. From left to right, representative immunoblot (showing two samples from 3xTgAD and two samples from 3xTgAD.TSPO^−/−^ mice) and protein quantification (normalized to ACTIN) for BACE1 (H), APOE (I) and IDE (J) (two-tailed unpaired t-test: *P* > 0.05). **K** mRNA quantification of *App* (2^-^(ΔΔCT)^ method; two-tailed unpaired t-test: *P* > 0.05). **L** soluble APPα/soluble APPβ ratio (two-tailed unpaired t-test: *P* > 0.05) in the whole hippocampus of 9-month-old mice

Immunolabeling of amyloid-β with 6E10 and 4G8 antibodies revealed a genotype effect depending on the age of the animals. Indeed, hippocampal pyramidal neurons showed a greater accumulation of 6E10 in the absence of TSPO at 9 months of age (Fig. 4A, B). A decreased intensity of 4G8 at 4 months-old, and an increase was measured in 3xTgAD.TSPO^−/−^ mice compared to 3xTgAD mice at 9 months-old (Fig. 4C). No extracellular Aβ deposits were observed in mice aged 2, 4 and 9 months, and granule neurons of the hippocampal dentate gyrus were also negative for 6E10 (see Fig. 3A). The intracellular staining of Aβ/APP antibodies seems to be mainly due to human Aβ/APP and not to mouse Aβ/APP, as WT mice of the same age (9 months old) clearly did not show any 6E10-ir and 4G8-ir (supp. Fig. 6). We then quantified the levels of amyloid-β in the parenchyma by separating the proteins solubilized in triton (Tx) and guanidine (Gu) which represent the non or poorly aggregated forms versus the more strongly aggregated forms, respectively. This revealed reduced amyloid-β accumulation in 3xTgAD.TSPO^−/−^ mice (Fig. 4D). Specifically, at the age of 9 months a decrease in Tx-Aβ42 was observed (−26 ±3.7%, *P* < 0.001) and Gu-Aβ42 was almost absent (−96 ±0.8%, *P* < 0.0001). Unlike Aβ42, which is the most toxic form of the Aβ, the accumulation of Aβ40 was not significantly affected by the absence of TSPO. To determine the mechanisms of the reduction in Aβ42, we measured the density of proteins involved in the production and degradation of amyloid-β. We quantified the levels of BACE1 (β-secretase), APOE (ApolipoproteinE) and IDE (insulin-degrading enzyme) by western blot but did not see differences between genotypes (Fig. 4H-J). In addition, *App* mRNA levels and the soluble APPα/soluble APPβ ratio were similar between the genotypes (Fig. 3K, L), suggesting that the decrease in Aβ42 was not due to a reduction in Aβ production.

**Fig. 5:**
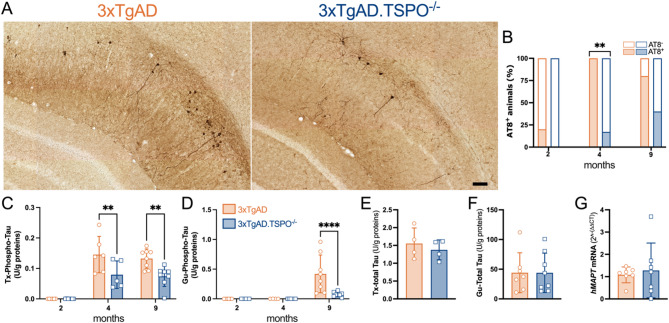
3xTgAD.TSPO^−/−^ mice exhibit reduced and delayed Tau pathology. **A** Representative example of AT8-ir in 9-month-old 3xTgAD and 3xTgAD.TSPO^−/−^ mice. Scale bar: 100µm. **B** Number of mice with AT8^+^ staining as function of the age (chi-squared, *P* < 0.01). **C-D** Quantification of Triton- (Tx, C) and guanidine- (Gu, D) soluble forms of pT231 Tau (two-way ANOVA, Tx-Tau: genotype x age, *F2,34* = 3.47, *P* = 0.04; genotype,* F1,34* = 13.84, *P* = 0.0007; age, *F2,34* = 41.56, *P* < 0.0001; Gu-Tau: genotype x age, *F2,32* = 6.07, *P* = 0.006; genotype,* F1,32* = 5.29, *P* = 0.028; age, *F2,32* = 12.47, *P* < 0.0001). **E–F** Quantification of Triton- (Tx, E) and guanidine- (Gu, F) soluble forms of total TAU in 9-month-old mice (two-tailed unpaired t-test: *P* > 0.05). **G** mRNA quantification of *hMAPT* in 9-month-old mice (2^-^(ΔΔ^.^CT)^ method; two-tailed unpaired t-test: *P* > 0.05)

We next examined the effects of TSPO knockout on phosphorylated-Tau levels, another hallmark of AD (Fig. 5A). AT8-ir showed a delay in the appearance of AT8^+^ neurons in the pyramidal cell layer (Fig. 5B). Observations showed that 4-month-old 3xTgAD.TSPO^−/−^ mice were less prone to express pathological forms of Tau (AT8^+^) (*P* = 0.009), a pattern partially preserved at 9 months of age compared to 3xTgAD mice (*P* = 0.09). The quantification of Tx-phospho-Tau 231 and Gu-phospho-Tau 231 levels confirmed the presence of a TSPO effect (Fig. 5C, D). A significant lower concentration of Tx-phospho-Tau forms at the ages of 4 (−45 ±11%, *P* < 0.05) and 9 (−44 ±8.9%, *P* < 0.05) months was measured. While the Gu-phospho-Tau forms appeared in 9-month-old in 3xTgAD mice, they were strongly reduced in age-matched 3xTgAD.TSPO^−/−^ (−82 ±3.4%, *P* < 0.0001). Importantly, the concentration of Tx-total-Tau, Gu-total-Tau and the *hMAPT* mRNA did not differ between 9-month-old 3xTgAD and 3xTgAD.TSPO^−/−^ mice (Fig. 5E-G), suggesting that the decrease in phospho-Tau forms is not a reflection of a decreased synthesis of Tau.

### TSPO is associated with GFAP in the hippocampus of 5xFAD mice

**Fig. 6:**
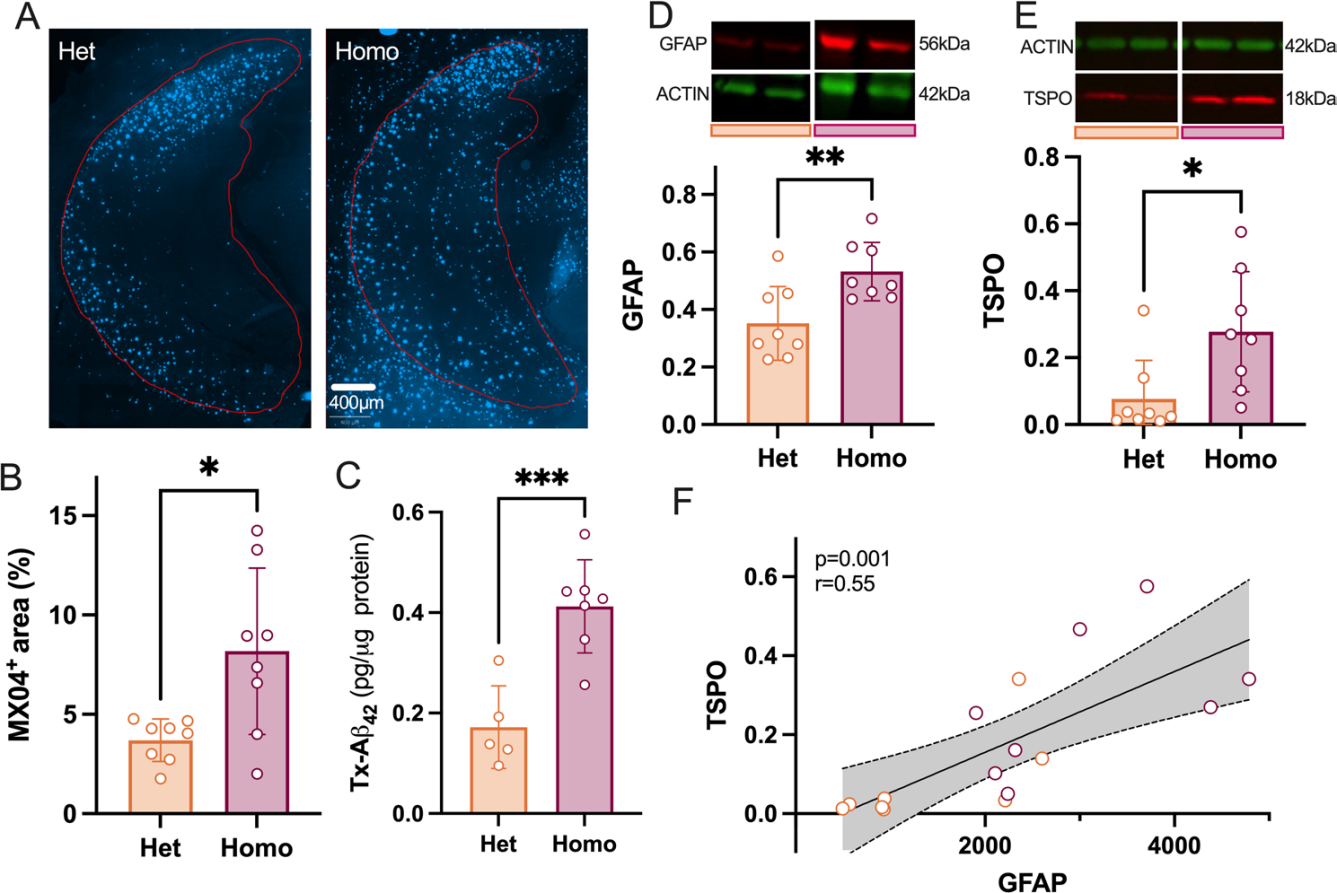
TSPO increase is associated with astrocyte reactivity in the hippocampus of the 5xFAD mice. **A** Representative example of MX04 staining in 5-month-old heterozygous (Het) and homozygous (Homo) 5xFAD mice. The red line underlines the hippocampus. Scale bar: 400µm. **B** MX04 density (% area) in the hippocampus (two-tailed unpaired t-test: *P* = 0.011). **C** Quantification of Triton-soluble forms of Aβ_42_ (two-tailed unpaired t-test: *P* = 0.0009). **D**-**E** Representative immunoblot (showing two samples from Het and two samples from Homo mice) and protein quantification (normalized to ACTIN) in the hippocampus of Het and Homo 5xFAD mice for GFAP and TSPO (two-tailed unpaired t-test: GFAP, *P* = 0.0077; TSPO, *P* = 0.018). **F** Correlation analysis (Pearson’s coefficient) between GFAP/ACTIN and TSPO/ACTIN ratios. Data are presented as individual values and mean ± SD (*n* = 8/group)

Finally, we used another AD model to further consolidate the link between astrocyte reactivity, TSPO density, and Aβ accumulation. To modulate Aβ levels in animals of the same age, we used homozygous and heterozygous 5xFAD male mice at 5 months of age. As expected, heterozygous animals showed less accumulation of amyloid plaques and Aβ_42_ (Fig. 6A-C). Importantly, heterozygous mice also showed reduced levels of both GFAP and TSPO (Fig. 6D, E). Moreover, as in humans (See Fig. 1R), TSPO directly correlated with GFAP levels (*r* = 0.55, *P* = 0.001, Fig. 6F). These observations – in another AD mouse model and in males—support the idea that TSPO density and astrocyte reactivity are linked to the progression of Aβ pathology.

### Glucose hypometabolism and oligodendrocyte dysfunctions in the hippocampus of aged 3 × TgAD mice

**Fig. 7:**
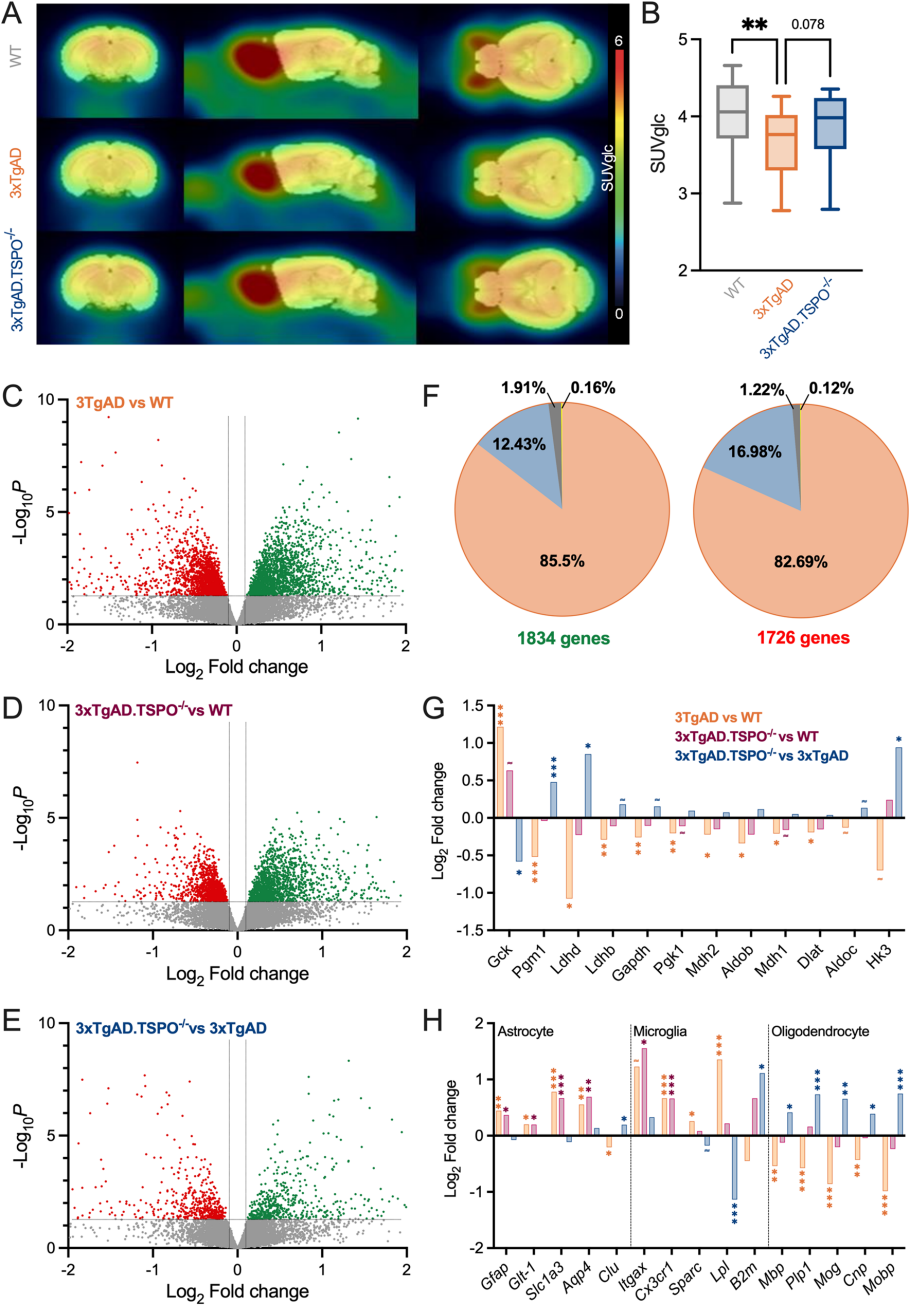
3xTgAD.TSPO^−/−^ mice reduced mRNA alterations. **A**-**B** Mean parametric images and quantification of [^18^F]FDG uptake (SUVglc) in WT, 3xTgAD and 3xTgAD.TSPO^−/−^ mice (n = 18/group; two-way ANOVA, genotype,* F2,969* = 6.07, *P* = 0.0024; brain area, *F18,969* = 10.7, *P* < 0.0001; genotype x brain area, *F36,969* = 0.109, *P* > 0.05). **C-E** Volcano plot of the differentially expressed genes in the hippocampus of 3xTgAD as compared to WT mice (**C**), 3xTgAD.TSPO^−/−^ vs WT (**D**) and 3xTgAD.TSPO^−/−^ vs 3xTgAD.TSPO (**E**). Dots represent individual gene whose the expression is downregulated (red), upregulated (green) or unaltered (grey, |log_2_FC|≥ 0.1 or *P* value > 0.05. (n = 5 animals/group). **F** Genes up- (left) and down-regulated (right) in 3xTgAD mice as compared to WT. Gene expressions were either specifically modified in 3xTgAD (orange), return to control levels in 3xTgAD.TSPO^−/−^ mice (blue), trend to return to control levels (grey) or their difference was accentuated in 3xTgAD.TSPO.^−/−^ mice (yellow). **G-H** Log_2_ Fold change in some genes involved in glycolysis (**G**) or cell type specific (H) (adjusted *P* value: *P* < 0.05; ≈: 0.05 < *P* < 0.1)

To gain a deeper insight into the alterations present in the AD model and the effects induced by the absence of TSPO, an analysis was carried out in 13- and 14-month-old old mice to determine whether the beneficial effects of TSPO are maintained when the pathology is well established. [^18^F]FDG accumulation (SUVglc) revealed a whole brain hypometabolism in 3xTgAD (*P* = 0.0014) that was partly reversed by the TSPO knock-out (*P* = 0.35 vs WT and *P* = 0.078 vs 3xTgAD, Fig. 7A-B).

Bulk mRNA sequencing was then used to quantify alterations in the hippocampus. Out of 12,929 genes analyzed, 3560 genes had altered expression in 3xTgAD compared with WT (1726 down-regulated and 1834 up-regulated), 2660 genes had altered expression in 3xTgAD.TSPO^−/−^ vs WT, and 982 genes in 3xTgAD.TSPO^−/−^ vs 3xTgAD (Fig. 7C-E). Interestingly, 68.83% of the genes modified in 3xTgAD.TSPO^−/−^ vs WT mice were also modified in 3xTgAD, (i.e. 51.43% of their modified genes compared to WT). Among genes with altered expression in 3xTgAD, 521 (17.5%) were normalized to control levels by the absence of TSPO, 56 (1.88%) showed the same trend, while the difference was greater for 5 genes (0.17%), and the same pattern applies whether the genes were up- or down-regulated in 3xTgAD mice (Fig. 7F). Using the Wikipathway 2024 database, Enrichr analysis using the Wikipathway 2024 database of the 577 genes whose expression was normalized by TSPO knock-out showed an involvement in « Glial cell Differentiation» (*Mag*, *Cnp*, *Plp1*, *Mbp*; Adj *p* = 0.010, OR: 45.19), and « Oligodendrocyte differentiation leading to myelin for CNS» (*Mag*, *Cnp*, *Mog*, *Plp1*, *Mbp*, *Sox10*; Adj. *p* = 0.042, OR: 8.49). Analysis with the MGI Mammalian Phenotype Level 4 2024 database showed « abnormal myelination» (*Gal3st1*, *Nkx6*-*2*, *Actl6b*, *Lgi4*, *Sox10*, *Mag*, *Gjc2*, *Gjb1*, *Mobp*, *Slc17a5*, *Plp1*, *Mbp*, *Ptpn6*; Adj *p* = 0.0062, OR: 5.57), and « abnormal myelin sheath morphology» (*Cldn11*, *Fa2h*, *Mag*, *Gjb1*, *Lgi4*, *Slc17a5*, *Plp1*, *Mbp*, *Cers2*; Adj *p* = 0.036, OR: 7.68) pathways. Genes involved in glycolysis show a trend towards decreased expression in 3xTgAD which was attenuated in 3xTgAD.TSPO^−/−^ (Fig. 7G). The expression of astrocyte and microglia genes was altered in 3xTgAD mice, and TSPO deletion modified the expression of some of these genes (Fig. 7H). Oligodendrocyte-associated genes altered in 3xTgAD were normalized by the TSPO knock-out (Fig. 7H).

### TSPO knockout protects against Tau build-up and Tau-induced cognitive deficits

**Fig. 8:**
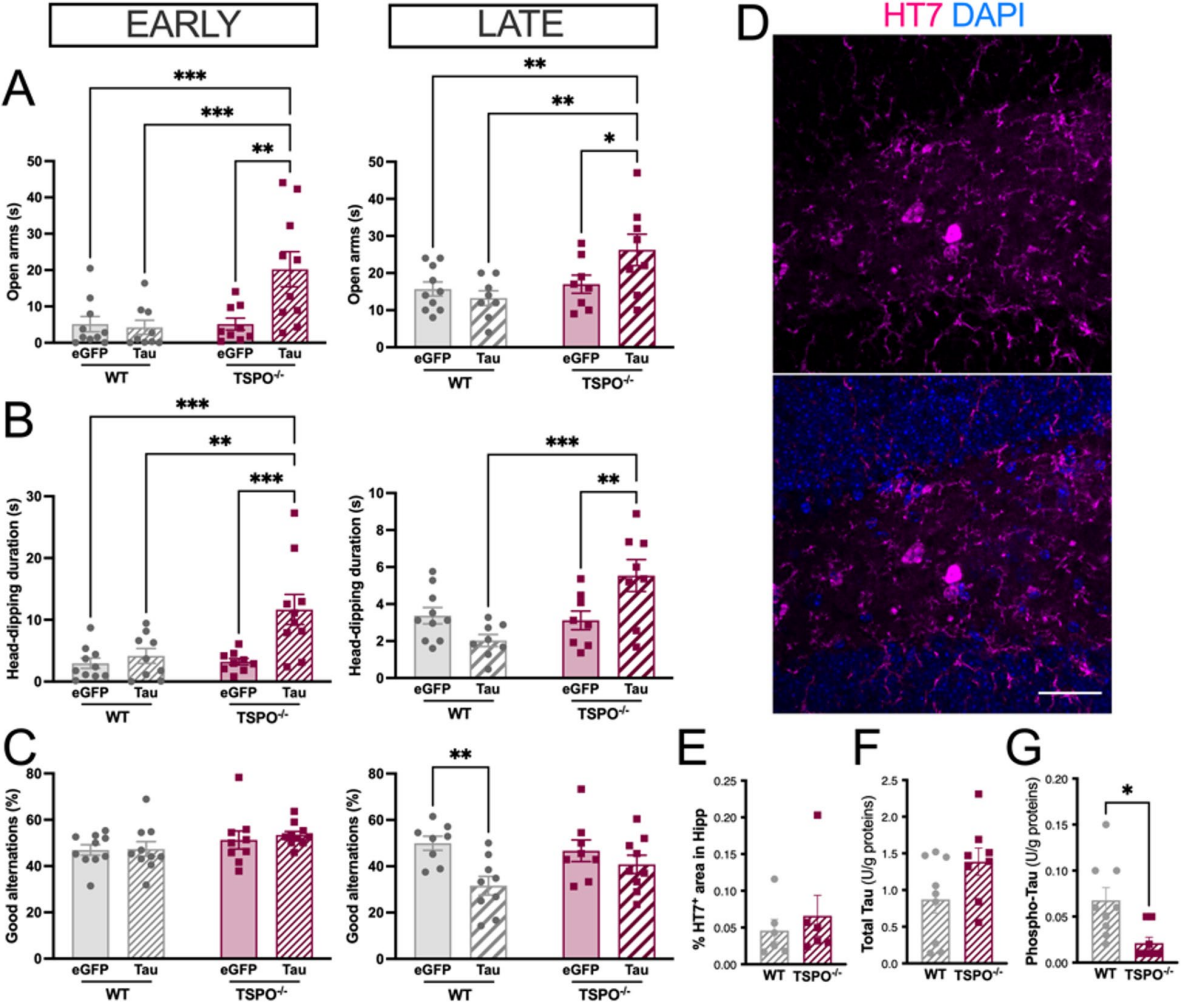
TSPO^−/−^ mice overexpressing Tau show reduced phospho-Tau and cognitive deficit. Early (1 month) and late (6 months) behavioral effects induced by the intra-hippocampal injection of adenovirus (Ad) encoding Tau or eGFP (control) gene. **A** Time past in open arms in the EPM at 1 month (left: two-way ANOVA, genotype x Ad, *F1,34* = 6.93, *P* = 0.012; genotype,* F1,34* = 6.96, *P* = 0.012; Ad, *F1,34* = 5.47, *P* = 0.025) and 6 months (right: two-way ANOVA, genotype x Ad, *F1,30* = 4.61, *P* = 0.04; genotype,* F1,30* = 6.88, *P* = 0.013; Ad, *F1,30* = 1.56, *P* > 0.05) post Tau induction. **B** Head-dipping duration in the EPM at 1 month (left: two-way ANOVA, genotype x Ad, *F1,34* = 5.9, *P* = 0.02; genotype,* F1,34* = 6.79, *P* = 0.013; Ad, *F1,34* = 10.4, *P* = 0.008) and 6 months (right: two-way ANOVA, genotype x Ad, *F1,30* = 11.26, *P* = 0.002; genotype,* F1,30* = 8.48, *P* = 0.007; Ad, *F1,30* = 0.93, *P* > 0.05) post Tau induction. **C** % of good alternation in the Y-maze at 1 month (left: two-way ANOVA, genotype x Ad, *F1,35* = 0.09, *P* > 0.05; genotype,* F1,35* = 3.4, *P* > 0.05; Ad, *F1,35* = 0.2, *P* > 0.05) and 6 months (two-way ANOVA, genotype x Ad, *F1,30* = 2.45, *P* > 0.05; genotype,* F1,30* = 0.56, *P* > 0.05; Ad, *F1,30* = 9.26, *P* = 0.005; two-tailed unpaired t-test: Ad-Tau in WT: *P* = 0.0028; two-tailed unpaired t-test Ad-Tau in TSPO^−/−^: *P* = 0.34) post Tau induction. **D** Representative example of Tau immunofluorescence at 6 months post Tau induction (top: Tau HT7 immunoreactivity; bottom: merge image with DAPI staining). Scale bar: 30 µm. **E** Quantification of % of positive HT7-ir area in the hippocampus of mice injected with the Ad encoding Tau (two-tailed unpaired t-test: *P* > 0.05). **F-G** Quantification of total Tau (F, two-tailed unpaired t-test: *P* > 0.05) and pT231 Tau (G, two-tailed unpaired t-test: *P* = 0.0104) demonstrating low values of pT231 Tau in TSPO^−/−^ mice

To evaluate if TSPO knockout directly affects Tau-related neuropathology and cognitive deficits, independently of Aβ pathology, we performed additional experiments. WT and TSPO^−/−^ animals received an intra-hippocampal injection of adenovirus (Ad) allowing the overexpression of Tau or eGFP for control. At 1- and 7-months post-Tau/eGFP induction, animals were tested for anxiety and spatial working memory to assess early and late effects. We observed a reduction of anxiety (exploration time of open arms, duration of head-dipping) in TSPO^−/−^ animals 1 month after the injection of Tau (Fig. 8A, B). In addition, Ad-Tau induced a late decrease in the % alternation in the Y-maze test in WT mice (−36.74±8.06%, *P* < 0.01), confirming a Tau-induced deficit in spatial working memory (Fig. 8C). Importantly, TSPO knockout protected from the deleterious effect of Tau (−12.56±8.42%. *P* > 0.05). *Postmortem* analysis validated the presence of Tau using HT7-ir (Fig. 8D). Importantly, no difference was observed neither in the % area covered by HT7-Tau nor in the Total-Tau concentration (Fig. 8E, F), indicating a similar transduction efficiency in WT and TSPO^−/−^ mice. In main contrast, the phospho-Tau concentration was decreased in the hippocampus of Ad-Tau treated TSPO^−/−^ mice, as compared to WT (Fig. 8G).

## Discussion

The use of TSPO as an in vivo marker of neuroinflammation in AD is widely reported in animal models and in humans [7, 11], but its functional role in the etiopathology remains largely unknown. First, we demonstrated in the human hippocampus that TSPO expression was not only increased in AD but also negatively correlated with glycolysis and positively correlated with neuroinflammatory markers. In addition, TSPO levels in astrocytes was impacted by the Aβ proximity. The importance of TSPO in AD was confirmed in the 3xTgAD mice model, where the absence of TSPO induced amelioration of glucose usage, Aβ and Tau markers, and reduced astrocytic reactivity. In the 5xFAD model, we showed that TSPO levels are also correlated with astrocyte reactivity. Finaly, in TSPO^−/−^ mice we demonstrated that Tau-induced cognitive deficits were inhibited as compared to WT, demonstrating the beneficial effect of TSPO reduction. The correlation between TSPO and AD markers (Aβ, Tau) in humans – and animal models [7]—and the reduction of Aβ and Tau density when TSPO is lowered suggest a cause-and-effect relationship and highlight the potential therapeutic impact of TSPO reduction. Thus, we showed that TSPO could represent a particularly interesting target for the treatment of AD.

In the human hippocampus, we observed a positive correlation between TSPO and markers of inflammation (*CD40*, *AXL*, *ABCA1* and *CSF1)* and astrocytes (*GFAP*, *GDPD2* and *SOX9*), suggesting a role of TSPO in astrocyte reactivity. ABCA1 in astrocytes was associated with increased phagocytosis activity and CSF1 with neurotoxicity [60, 61], suggesting a role for TSPO in astrocyte reactivity. The increase in TSPO in *APOEε4* carriers (independently of the AD phenotype) supports the hypothesis that TSPO plays a role in neuroinflammation, as *APOEε4* primes astrocytes for a pro-inflammatory state [62, 63]. Neuronal cultures treated with conditioned media from APOE4 astrocytes did not demonstrate a divergent response in comparison to conditioned media from APOE3 astrocytes, unless the astrocytes had been exposed to Aβ [63]. This observation underscores the significance of Aβ in the context of the impact of APOE4. The reduction of astrocyte reactivity in the hippocampus of 3xTgAD.TSPO^−/−^ mice (as shown by reduction in specific gene expression, CLU and GFAP density) showed a direct link between TSPO and astrocytic reactivity. Similar results were obtained with TSPO ligands in an experimental model of high-salt neurotoxicity, sustaining our conclusions [64]. Because in the human AD brain the TSPO density in GFAP positive area is increased near Aβ deposits, it might be suggested that TSPO increases are linked to the presence of Aβ. In addition, analysis of GFAP as a function of age and hippocampal area provided a greater level of detail. The absence of differences at 2 months between 3 and 3xTgAD.TSPO^−/−^ mice, and at 9 months between WT and TSPO^−/−^, indicates that the reduced astrocyte reactivity observed in 9-month-old 3xTgAD.TSPO^−/−^ mice compared to 3xTgAD is not innate Furthermore, astrocyte reactivity in 3xTgAD mice was greater in the subiculum and dorsal hippocampus vs. ventral hippocampus, corresponding to areas of greatest Aβ and TSPO accumulation [47]. These observations support the correlation between TSPO and GFAP observed in both 5xFAD mice and humans, i.e. TSPO and GFAP levels increasing proportionally. Moreover, inhibiting TSPO in 3xTgAD mice reduced astrocyte reactivity and Aβ pathology, while reducing Aβ pathology in 5xFAD mice led to a decrease in astrocyte reactivity and TSPO levels (see Fig. 6). These findings suggest that TSPO expression, astrocyte activation, and Aβ pathology may participate in positive feedback loop amplifying neuropathological processes.

The alteration in astrocyte morphology could also be indicative of either a low reactivity or astrocyte atrophy. Indeed, a reduction in the complexity of astrocytes has been described in the dentate gyrus of 3xTgAD mice at the age of 6 months and in CA1 at the age of 18 months, in parallel to the development of hypertrophic astrocytes [65]. In other studies, hypertrophy of CA1 astrocytes of 3xTgAD mice was observed at the age of 8–9 months [66, 67]. As the reduction of astrocyte complexity in 3xTgAD.TSPO^−/−^ mice is associated with a decrease of pathological markers (Aβ, Tau) in our study, it is more likely that this morphological modification reflects a lower reactivity level in 3xTgAD.TSPO^−/−^ mice as compared to 3xTgAD mice, rather than atrophy. Reactive astrocytes are regularly observed in other pathologies, such as in mouse models of multiple sclerosis [31]. The knockout of TSPO induced a lower astrocyte reactivity and decreased the severity of symptoms in response to the experimental induction of multiple sclerosis [24], thereby supporting the pro-inflammatory hypothesis of TSPO in different pathological contexts. A reduction in astrocyte reactivity caused by other genetic modifications has also been shown to have ameliorative effects in AD and amyotrophic lateral sclerosis [66, 68, 69]. It is important to note that the activation of astrocytes is observed at early disease stages of AD^16, 17, 18^ and overexpression of TSPO by astrocytes has been observed in other AD models like in TgF344-AD rats [16–18, 70]. As astrocytes participate in the amplification of the pathology by stimulating the synthesis and spread of Aβ and Tau [71–74], our data support the hypothesis that TSPO in astrocytes could be one of the early determinants of the disease progression in AD and, by extension, of some other neurodegenerative pathologies.

TSPO density is also associated with decreased expression of genes involved in glycolysis. The impact of TSPO on glucose consumption has already been hypothesized in response to LPS [25]. Our results show that the absence of TSPO mitigates glucose uptake deficits and positively influences glycolysis pathways, reinforcing the connection between TSPO and glucose metabolism. Among the proteins involved in glycolysis, ALDOC is predominantly expressed by astrocytes and both its density and activity are decreased in AD, leading to a decrease in glycolysis and an increase in gluconeogenesis [75–77]. In accordance with these observations, our data show a decrease in *ALDOC* mRNA in the human hippocampus and we demonstrate that the absence of TSPO in 3xTgAD.TSPO^−/−^ mice increases ALDOC protein levels. Thus, the reduction in glucose uptake seen in 3xTgAD mice and then the beneficial effect of the lack of TSPO could be at least partly related to astrocyte activity. It may be pointed out that ALDOC is not a limiting enzyme in glycolysis, but that its reduction in AD may contribute to a drop in the production of products of glucose catabolism. A decrease in FDG uptake has already been reported in 3xTgAD mice where the authors have shown a dysregulation of the serine pathway [49]. The absence of alteration in PSAT1 and PHGDH protein density did not confirm this observation. However, we showed that 6 of the proteins whose levels increased in 3xTgAD mice had densities inversely correlated with TSPO in human, assuming common mechanisms between the animal model and humans.

At the early stages used herein, we observed an impact of the absence of TSPO on astrocytes but not on microglia. The absence of TSPO-induced microglia changes resembles the absence of difference between TSPO^−/−^ and WT mice in response to neuronal injury [48]. A role for microglial TSPO at later stages of AD pathology may be hypothesized, as it appears essential in other contexts. For example, it has been reported that the cellular effects of diazepam are modulated through microglial TSPO [22]. A difference in temporal course between TSPO from astrocytes and TSPO from microglia has already been observed in various pathologies [31] and points to the fact that the pathological stage must be considered in the development of therapeutic strategies. Considering the early stage of the pathology in our study, i.e. the absence of CD68 (marker of activated microglia), of STAT3 (marker of the reactivity of astrocytes), of extracellular Aβ-deposits and of cognitive effect, it can be hypothesized that microglia TSPO from microglia is not a key player as astrocyte TSPO in early AD. Microglia TSPO may play an important role later in the pathology, as shown by microglia TSPO in the human brain being mainly associated with microglial expansion and CD68 expression [12, 78]. In line with this idea, gene expression analysis in aged mice showed that the absence of TSPO had an impact on the expression of microglial genes affected in 3xTgAD mice. Nevertheless, our data from the human hippocampus do not support a direct contribution of microglial TSPO in AD. In addition, oligodendrocytes were also suspected to play roles in AD [79], and TSPO is involved in oligodendrocyte functions [80]. This may explain why oligodendrocyte gene expression is affected by TSPO inhibition, suggesting that oligodendrocyte TSPO could also contribute to AD. Our observations demonstrate that the impact of TSPO on cell types during AD is a dynamic phenomenon, and that the timing of the observation is fundamental. Given that the current data are limited to mRNA expression, additional studies will be needed to evaluate the functional impact of TSPO on oligodendrocytes, which falls outside the scope of this work.

Compared with 3xTgAD mice, 3xTgAD.TSPO^−/−^ mice displayed lower intracellular Aβ levels at 4 months of age. At 9 months, they showed higher intracellular Aβ levels but reduced soluble and aggregated forms of Aβ. Since intracellular Aβ is known to serve as a source of extracellular Aβ, with intracellular staining decreasing and extracellular deposition increasing as pathology progresses [2, 81], these findings suggest a delay in the onset of amyloid pathology in 3xTgAD.TSPO^−/−^ mice.At 9 months of age, a stage before extracellular Ab-plaque deposition, there was an increase in the intracellular staining of Ab in 3xTgAD.TSPO^-/-^mice as revealed by 6E10-ir. In contrast, we reported a decrease in poorly and highly aggregated Ab42 forms. This observation could reveal a delay in the onset of the pathology, as it has been shown that the intracellular Ab increase appears before the extracellular one[81]. In addition, the direct consequence of the accumulation of sAPPa being a limitation of the amyloidogenic maturation of APP[82], increase in sAPPa in 3xTgAD.TSPO^-/-^mice may further reduce the release of Ab. However, As the density of BACE1 remained unchanged, its can indicate a partial preservation of the Aβ synthesis pathway, unless BACE1 activity was reduced. To assess if BACE1 activity was modified, the sAPPα/sAPPβ was estimated but did not show a significant effect. No effect of TSPO knockout was observed on ApoE or IDE levels, proteins involved in Aβ clearance and degradation, respectively. However, other mechanisms could come into play, as for example, the phagocytosis capacities of glial cells, the first key element in the degradation of Aβ. These observations and hypotheses will need to be validated in studies conducted at later stages of the disease, when Aβ plaques are present. However, the primary aim of the present study was to investigate the role of TSPO at a very early stage—specifically, before plaque formation.

Another striking finding concerns Tau-related pathology. In two different models, we showed that the absence of TSPO limits the burden and the functional consequences of Tau pathology. Since the Tau pathology correlates to the cognitive deficits observed in AD patients [83, 84], our observation of decreased Tau and Tau-induced cognitive deficits suggests a strong translational potential of inhibiting TSPO. Indeed, the exogenous overproduction of Tau induced higher densities of phospho-Tau and a higher cognitive impairment in the presence of TSPO. Importantly, TSPO^−/−^ mice injected with the Tau vector displayed the same level of total Tau but a reduction in phospho-Tau and no cognitive impairments, as compared to WT injected mice. This suggests that the absence of TSPO limits Tau phosphorylation which would in turn prevent cognitive loss. The exact mechanism of action leading to this reduction in conversion of Tau into pathological forms should be the subject of future analysis. In an observational study, the accumulation of TSPO was shown to be correlated with that of phospho-Tau in P301S mice [85], in support of our results. However, the decrease in anxiety levels observed in Ad-Tau TSPO^−/−^ mice may represent a side effect and warrants further investigation to be fully understood.

Thus, our study with genetic inhibition of TSPO demonstrated a pro-inflammatory and deleterious role of TSPO in the appearance of neurochemical markers of the pathology. Other studies have shown that TSPO antagonism in 3xTgAD mice or in LPS-treated mice limited Aβ load expansion, thus supporting our conclusions [27, 28]. Another study in 3xTgAD mice also reported a reduction in Aβ but with the use of a TSPO agonist [26]. In other pathologies, such as traumatic brain injury, stroke or Parkinson’s disease, the use of agonist and antagonist ligands of TSPO exhibited beneficial effects [86–88], making the simple antagonist/agonist definition insufficient to understand the effects of TSPO ligands. Dual dose effects of TSPO antagonists were also observed in cell culture [89]. TSPO exists in monomeric and complex multimeric forms [90]. It has been shown that the presence of a TSPO antagonist induced an increase in the ratio between the 36 and the 72 kDa TSPO polymers [89]. However, as the function of TSPO may be linked to its multimerization [90–92], it would be interesting to determine the impact of the treatments used in previous studies on the complexity of TSPO forms to better understand the biological effects of TSPO agonists/antagonists. A 2-year follow up clinical study suggested that a relatively preserved level of TSPO is associated to a favorable cognitive outcome compared to patients with increased TSPO [93–95]. In addition, high levels of TSPO are mostly associated with elevated cognitive deficits [7, 96–100]. All these data indicate that TSPO function per se may be an interesting therapeutic target, while further research is warranted to clarify if its inhibited or enhanced of its function in a disease-specific context is the most beneficial option.

Interestingly, both animal models and human AD brain samples displayed an increase in reactivity markers of both astrocytes and microglia [9, 101–104] which could suggest a similar implication of neuroinflammation across species. Genome-wide association studies (GWAS) and single-cell RNA sequencing studies strongly suggested that glial cells are causally involved in the AD pathology [6, 105–109]. In addition, our data, showing a reduction of pathological markers when TSPO levels are lowered, are consistent with observations in humans, where higher TSPO levels are associated with greater cognitive impairment [7, 96–100]. In our 3xTgAD experiment, we only used females as pathological AD markers are increased in females in mouse AD models [110–118], demonstrating a higher vulnerability of female mice. In human, sex differences are widely reported, and the AD prevalence is significantly higher in elderly women than in men [119–124]. In addition, amyloid-β deposits are more extensive throughout the brain in women than in men in the first neurofibrillary stages, and amyloid-load and phosphorylated Tau are denser in women [125–128], demonstrating a higher vulnerability to AD in women. Thus, we only focused on highly vulnerable females but, further studies will be needed in males to determine the sex effect of TSPO inhibition. In addition, the data presented here demonstrate a beneficial effect of the absence of TSPO in the early stages of the disease and on Tau-induced cognitive deficits. Further complementary analyses at later stages will allow us to complete our observations. In addition, correlations based on protein and mRNA values from the human hippocampus have inherent limitations, including possible effects of postmortem interval, clinical confounders, and cellular heterogeneity. Nevertheless, they provide unique insights into molecular alterations in the human brain, and our conclusions are supported by converging human and mouse data.

Our data show that TSPO is expressed by astrocytes, but not uniformly. Its overexpression is associated with astrocyte reactivity and correlates with GFAP. It is particularly increased in the vicinity of plaques, suggesting a role linked to astrocyte reactive state rather than ubiquitous expression. However, not all astrocytes seem to respond in the same way, since TSPO does not correlate with another reactivity marker, S100A. These differences, combined with the heterogeneous distribution of pathology in the hippocampus, could explain why the impact of TSPO on astrocyte reactivity, assessed by morphological analysis, varies depending on the region studied.In addition, we observed a reduction in glucose uptake in 3xTgAD mice and an increase in GAPDH activity, both of which were normalized in the absence of TSPO. Furthermore, TSPO deletion led to an increased density of glycolytic enzymes in mice, and, in humans, TSPO levels were negatively correlated with these enzymes. Collectively, these findings suggest a reduction in glycolysis in AD, associated with a compensatory increase in GAPDH activity. Notably, the absence of TSPO mitigates these alterations. Thus, TSPO appears to regulate astrocytic glycolysis through a mechanism that remains to be elucidated. Its effect on Aβ deposition could therefore result from modulation of glycolysis as inhibition of glycolysis has been shown to increase Aβ accumulation and Tau phosphorylation [129–131]. An increase in Aβ would, in turn, increase its negative impact on glycolysis and stimulate astrocyte reactivity [129, 132], thereby reinforcing the negative effect of TSPO. Within this framework, the effects of TSPO would be most evident under conditions of overexpression, when astrocytes are reactive, whereas its role in glycolysis would be limited under physiological conditions. This could potentially explain the absence of differences in astrocyte reactivity between WT and TSPO^−/−^ mice. The role of TSPO in other cell types appears to be less significant, but only future studies, for example using conditional knockout of astrocytes or other cells, and the use of TSPO^−/−^ mice to analyze glycolysis will be able to firmly validate our hypotheses.

In conclusion, this study establishes a link between increased TSPO and worsening AD pathology (Aβ and Tau) and is supportive for a role of TSPO in decreasing glycolysis and increasing astrocytic reactivity in the human hippocampus. TSPO depleted 3xTgAD mice data support these observations by showing a reduction in Aβ, Tau, astrocytes reactivity and a stimulation of glycolysis, at pre-plaque stages. These functions of TSPO could explain the increase in cognition in response to stimulation of astrocytic glycolysis and the increase in the accumulation of Aβ by inhibition of astrocyte glycolysis [129, 133]. In line with previous reports, the present study provides novel findings demonstrating that modifying astrocyte function constitutes a therapeutic intervention, even in the absence of effects on microglia [66, 134].

## Materials and methods

### Animals and sample collection

Experimental approval was obtained from the ethics committee for animal experimentation of the canton of Geneva, Switzerland. All the animals were reared under standard light/dark conditions with ad libitum access to food and water, and randomly assigned and tested blind to both experimental conditions and genotypes. Details of strains and euthanasia are given in supplemental methods.

### Postmortem human samples

Experimental approval was obtained from the local ethics committee for human experimentation of the canton of Geneva, Switzerland. Human frozen hippocampus were obtained from the Netherland brain bank and formalin fixed paraffin embedded human hippocampus Sects. (20 µm) from the Geneva Brain Bank [135]. Characteristics of the samples are given in supp. Table 1.

### Protein extraction and quantification

Human and mouse samples were sonicated after immersion in a solution of triton (50mM Tris HCl, 150mM NaCl, 1%Triton × 100, protease and phosphatase inhibitors 1x, pH = 7.4). After centrifugation (20 000 g, 20 min, 4 °C), the supernatants collected contain the triton (Tx)-soluble fraction. The pellet was taken up with a solution of guanidine (5M guanidine, 50mM Tris HCl, protease and phosphatase inhibitors 1x, pH = 8). After gentle agitation (3h, 4 °C) and centrifugation (20 000 g, 20 min, 4 °C), the supernatants collected contain the guanidine (Gu)-soluble fraction. A protein assay was performed using the BCA kit to quantify the total amount of proteins. The determination of the amount in different forms of Aβ40, Aβ42, sAPPα, sAPPβ, and Tau was carried out by ELISA using commercial kits (see supplemental methods for details).

### Immunostaining and Sholl analysis

Hippocampus sections were treated with primary and secondary antibodies before Sudan black treatment and DAPI counterstaining or DAB revelation. Detailed protocols and the list of antibodies are given in supplemental methods. Images of entire sections were acquired using Zeiss Axioscan for measurement of % of labeled area and fluorescence intensity. The regions of interests have been drawn manually based on anatomy using ImageJ software. The number of animals presenting AT8^+^ neurons in the hippocampus was manually determined. For the Sholl analysis, confocal images (2 µm steps) were analyzed using the ImageJ software plugging. The soma of cells was manually drawn, and concentric circles were automatically applied (2 μm distance between circles) to measure the number of intersections along the Sholl radii, the area under the curves and the size of soma.

### Colocalization

Confocal images were acquired using the Axio Imager.Z2 Basis LSM 800 microscope (Zeiss) with × 40 objective and at a rate of 1 image by 0.21 µm or using a × 10 objective and at the rate of 1 image by 2.24 µm. Figures show the maximum intensity projection (Z-stack Processing, ImageJ). In the region of interests, the area fraction occupied by each marker and their overlapping fraction were quantified from binary images obtained through thresholding in ImageJ.

### APOE genotyping

APOE genotyping was obtained using a TAQMAN-based Real-Time PCR assay using a combination of both SNP rs429358 (Cys130Arg) and rs7412 (Arg176Cys) using commercial reagents and following manufacturer instructions (TaqMan SNP genotyping assays, Assay ID 3084793_20 & 904973_10, ThermoFisher Scientific, Waltham, MA, USA), on DNA extracted from brain samples using DNeasy Blood & Tissue Kit (Qiagen, Hilden, Germany).

### Western blot

Triton-soluble proteins were treated for western blot analysis using a LF-PVDF membrane. Detailed protocols and the list of antibodies are given in supplemental methods. Densitometry analysis using ImageJ was performed to quantify proteins and raw data were expressed as function of ACTIN.

### Proteomic

Triton-soluble proteins were precipitated and digested with trypsin. Peptides were analyzed by nanoLC-MSMS using an easynLC1200 (Thermo Fisher Scientific) coupled with an Orbitrap Fusion Lumos Mass Spectrometer (Thermo Fisher Scientific). Data analysis was performed with Spectronaut (Biognosys) using the mice reference proteome database (Uniprot). Both peptide precursor and protein FDR were controlled at 1% (Q value < 0.01) and data were validated with at least 2 unique peptides per protein. Proteins were considered to have significantly changed in abundance with an FDR ≤ 0.05 and an absolute fold change log2FC ≥|0.1|.

### Analysis of altered pathways and cell origin identification

Differential density analysis was performed in mouse protein using the Limma package in R. Functional enrichment analysis was carried out using the R studio with the GO biological processes database based on genes whose expression is significantly correlated with TSPO (human data) or based on genes identified by differential density analysis (mouse data). The identification of cell expression was performed using either the expression weighted cell type enrichment (EWCE) method in mouse samples or the human protein atlas database in human samples.

### mRNA sequencing and qPCR

Total RNA was extracted using the RNeasy mini (mice) and micro (human) kits (Qiagen) and cDNA synthesis were performed using the SuperScript VILO cDNA synthesis kit (Invitrogen) according to manufacturer instructions. Quantitative PCR were performed using PowerUp^TM^ SYBR^TM^ Green (Applied Biosystems) detection and PCR cycles as advice by the manufacturer. Primers used for the detection of mRNA were indicated in supplemental methods. Relative mRNA expression was normalized to *Gapdh* or *hPPIA*. Bulk mRNA sequencing was performed on an HiSeq 4000 (Illumina). Data was processed using the rna-seq nextflow pipeline [136]. The DGE analysis was performed using the limma-voom method from the limma R package [137] contained in the edgeR pipeline [138].

### mRNA analysis with the nanostring nCounter analysis system

Raw RNA data from the inflammation panel containing 770 RNA probes (NanoString) has been previously published [139]. TSPO/ACTIN was measured on the same samples and Pearson’s correlations between RNA levels and TSPO were researched.

### In situ enzymatic redox activity

Hippocampus sections were treated for in situ enzymatic redox activity of major metabolic pathways using a quantitative enzymatic histo-biochemistry approach with the nitro blue tetrazolium (NBT) assay. Detailed protocols and buffer compositions are given in supplemental methods. Brightfield images were acquired using a Zeiss Axio Scan Z1 scanner with a 20 × objective. Quantification of in situ enzymatic activity was performed using QuPath software where brightfield images were first converted to grayscale with intensity values set between 0 (lowest) and 2 (highest). Then, the optical density of the NBT signal was determined on a per-pixel basis within the regions of interest based on anatomy.

### [^18^F]FDG uptake

The mice were anaesthetised for [^18^F]FDG injection (5.47 ± 0.33 MBq) and then awakened for 40 min of accumulation. Under anaesthesia, a static PET acquisition of 3 mice simultaneously was performed 40 to 60 min after injection following a CT image. Body temperature was monitored and maintained using a rectal probe and heated bed. Images were acquired on a FLEX TriumphTM preclinical PET-CT scanner (Gamma Medica-Ideas, Nortridge, CA). Using PMOD (v4.401, PMOD Technologies LLC), [^18^F]FDG-PET images were spatially normalised to an [^18^F]FDG-PET template, and mean parametric images were expressed as standard uptake values corrected for blood glucose, injected dose and weight (SUVglc)_._ Images were analyzed using the VOI template integrated to PMOD to extract the radioactivity from each brain VOI [140].

### Brain Adenovirus (Ad) injections

Under isoflurane anesthesia and buprenorphine (Temgesic), a bilateral stereotaxic injection of 2 µl of Ad-Tau (3.10^10^ IFU/ml) or Ad-GFP (1.10^10^ IFU/ml) was performed into the dorsal hippocampus (AP: −3mm, Lat: ± 3mmm, V:−1.5 mm). After injecting at a rate of 0.2 µl/min, the syringe was left in place for 2 min before withdrawal. Ad-Tau was prepared in the laboratory according to the protocol described previously [89]. Briefly, the complete human Tau sequence was cloned and then inserted into pAd/CMV/V5-DEST adenoviral vectors, under the control of a cytomegalovirus promoter (ViraPower Adenovirus Expression System, Invitrogen). The Ad-Tau was verified by sequencing and the production of Tau by western blot. After titration, the viruses were stored at −80 °C before their use. Before injection, they were diluted in 0.1 M phosphate buffer saline (PBS) with 0.001% pluronic acid.

### Statistics

A sample size analysis was performed using the graphical Douglas Altman's nomogram approach with p ≤ 0.05 and β < 0.2 considering phosphorylated-Tau as the main outcome. In a previous study using the same detergents for protein extraction, the same pT231Tau ELISA kit and 3xTgAD mice, a variability of less than 6% on average was observed [141]. Thus, for the purpose of demonstrating a difference of at least 15% between 3 and 3xTgAD.TSPO^−/−^ mice, 6 animals per group were sufficient. We used 8 animals and observed a difference of 82%. Animals were randomized for virus injection. Data production and analysis of raw data of all techniques was done blind to the identification of the samples. For each group of measured variables, outliers were identified using the ROUT method (maximal false discovery rate = 1%) and excluded of the statistics. No animal was systematically removed and the number of individual observations per group is indicated in each figure. Comparison between two groups were performed using t-test. The number of mice AT8^+^ was compared using chi-squared. One- and two-way ANOVA were used to analyze the effect of AD stage in human, genotype and age in mice. Kruskal–Wallis test with the Dunn’s multiple comparisons post hoc test was used to analyze the human TSPO density and gene expression. Data are presented as individual values and mean ± SE. All statistical experiments were performed with GraphPad Prism 9. The number of stars on graph indicate the level of significance, as follows: *p < 0.05, **p < 0.01, ***p < 0.001, ****p < 0.0001. Correlation analyses were tested with the Pearson’s coefficient.

## Supplementary Information

Supplementary Material 1.

## Data Availability

The datasets generated during and/or analyzed during the current study are available from the corresponding author on reasonable request.

## Abbreviation

TSPO: 18KDa translocator protein
