# Supplementary material for: TSPO contributes to neuropathology and cognitive deficits in Alzheimer’s disease
Source: J Neuroinflammation. 2025 Oct 28;22:248. doi: 10.1186/s12974-025-03583-4 (PMC12570730; doi:10.1186/s12974-025-03583-4)
Supplement: Supplementary file 1 — Supplementary Material 1. [file 12974_2025_3583_MOESM1_ESM.pdf]

Supplemental methods

Animals and sample collection

A deletion of exon 2 and 3 of the *Tspo* gene made it possible to generate TSPO$^{-/-}$ mice on a C57BL/6 genetic background. The identification of knockout mice was done by PCR using genomic DNA: the amplicons are 489-bp in size for the wild-type allele and 246-bp for the knockout allele. The complete absence of TSPO protein has been verified using western blot. The 3xTgAD C57BL6/129SvJ hybrid background mouse model was backcrossed for more than 25 generations onto C57BL/6. Resulting C57BL/6-3xTgAD mice were genotyped to validate the presence of APP_{SWE}, Tau_{P301L} and $PS1_{M146V}^{+/+}$. C57BL/6-3xTgAD and C57BL/6-*Tspo*$^{-/-}$ mice were crossed to generate the APP_{SWE}, Tau_{P301L}, $PS1_{M146V}^{+/+}$ and TSPO$^{-/-}$ model. Female WT, TSPO$^{-/-}$, 3xTgAD and 3xTgAD.TSPO$^{-/-}$ mice were reared under standard light/dark conditions with *ad libitum* access to food and water. Heterozygous and homozygous 5xFAD male mice harboring the mutant human APP (Swedish: K670N/M671L, Florida: I716V, London: V717I) and PSEN1 mutations (M146L, L286V mutations) under the presence of mouse Thy1 promoter, were reared under standard light/dark conditions with *ad libitum* access to food and water. Animals were randomly assigned and tested blind to both experimental conditions and genotypes. Under anesthesia, an intracardiac saline perfusion was performed before brain removal. One hemisphere was immersed in formalin for 24 hours and then in a sucrose solution of increasing concentration before being frozen and cut with a cryostat (30 µm sections) for carrying out immunohistochemistry and immunofluorescence. The other hemisphere was dissected to isolate the hippocampus for protein measurement and the cortex for mRNA measurement. The samples were frozen in liquid nitrogen and then stored at -80°C.

ELISA

The determination of the amount in different forms of Aβ40, Aβ42, sAPPα ,sAPPβ, and Tau was carried out by ELISA from the following kits: Aβ40 human ELISA kit; Aβ42 human ultrasensitive ELISA Kit and Tau (phospho) [pT231] human ELISA Kit from Thermo Fisher, and sAPPα and sAPPβ ELISA Kit from Mybiosource. The procedure was performed according to the instructions of the kits. Briefly, the samples diluted in the dilution buffer were exposed for 2-3 hours to the specific antibody attached to the wells of the 96-well plate. After washing, the primary and then the secondary antibody were introduced into the wells for 30 min before adding the stabilized chromogen (30 min) and the stop solution. The OD reading was performed at 450 nm in the presence of a standard curve, specific to each kit. Data are expressed as a function of the total amount of proteins.

Immunostaining

Hippocampus sections (4 slices separated by around 200 µm/animal; 4 slices per AD subjects) were incubated with the primary antibody (4°C, overnight), rinsed in 1xPBS, incubated with the secondary antibody (90min, RT), rinsed in 1xPBS and treated with Sudan Black (0.3% in 70% ethanol) before being stained with a DAPI (30 nM, 10min) or Methoxy-XO4 (MxO4) solution (1/500, 30 min, Tocris). To increase the detectability of the AT8 staining, a revelation with 0.2 mg/ml DAB (Sigma-Aldrich) in 1xPBS containing 100 µl/L H_2O_2 was performed instead of Sudan black and DAPI staining. The procedure was modified to co-stain STAT3 (Cell signaling) and VIMENTIN (Abcam) as follows: the sections were pretreated with methanol (-20°C, 20 min), rinsed in 1xPBS and then immersed in a blocking solution (1xPBS, 0.3% Triton, 1% BSA) before being shaken for 3 days with the antibody solution (1/200 in Signal Stain). After two series of rinsing in 1xPBS interspersed with exposure to the

secondary antibody (1/200 in blocking solution), the sections are mounted using Fluorsave and marked with DAPI (30 nM, 10min). The antibody list was the following: 6E10 (1/200, mouse, Biolegend), 4G8 (1/500, mouse, Biolegend), AT8 (1/1000, mouse, ThermoScientific), GFAP-Cy3 (1/1000, Sigma), HT7 (1/500, mouse, Invitrogen), IBA1 (1/300, Rabbit, Wako), TSPO (1/150, Rabbit Ab100907, Abcam, in human brain and in mice for the co-staining with IBA1, and 1/150, Goat Ab118913, Abcam, in mice for the co-staining with GFAP), rabbit anti-mouse HRP (1/100, Dako), goat anti-rabbit Alexa Fluor 488 (1/200, Invitrogen), chicken anti-goat Alexa Fluor 594 (1/200, Invitrogen) and goat anti-mouse Texas Red (1/200, Invitrogen).

Western blot

Twenty µg of proteins were denatured in 1x Laemmli buffer, 2-5% b-mercapto-ethanol for 10min at 70°C and then migrated with protein ladder (Precision plus protein all blue standards, Bio-Rad) in Bio-Rad gels (Criterion TGX) at 150V for 55min with the manufacturer's protocol (Bio-Rad). Transfer on LF-PVDF membrane was performed for 7min at 2.5A constant, and up to 25V in the manufacturer buffer using the trans-blot turbo transfer system (Bio-Rad). Membrane was saturated in Tris-buffer (20 mM Tris, 150 mM NaCl, 0.1% tween, pH=7.4) containing 5% non-fat dry milk for 45min and then incubated 48h at 4°C with the primary antibody in Tris-buffer containing 5% non-fat dry milk. Following 3 washes in Tris-buffer (10 min, RT), the membrane was immerged in the secondary antibody in Tris-buffer containing 5% non-fat dry milk for 90 min. Following 3 washes in Tris-buffer (10 min, RT), the fluorescence was detected using the iBright imaging system (ThermoFisher Scientific). The same membrane was reused after a new saturation step. Primary antibodies used at 1/250 were as follows: Actin (Sigma), ApoE (Abcam), BACE1 (Cell signaling), Clusterin (Santa Cruz), GFAP-Cy3 (Sigma), IDE (Abcam), TSPO (Abcam) and secondary antibodies (1/1000) were Alexa Fluor antibodies from Thermo Fisher.

qPCR

Primers used for the detection of mRNA were designed as indicated in the following table:

**Gene name**	**Forward**	**Reverse**
hALDOC	CACTCAATGCCTGGAGAGGACA	CGCCATCTCCACTGCCTTCATA
hAMIGO2	CAGAACGGGAAAGTCAGGCT	CGCCACAAAAGGTGTGTCAG
hSL100A10	TCGCTGGGGATAAAGGCTAC	AAGAAGCTCTGGAAGCCCAC
hPPIA	TTCATCTGCACTGCCAAGAC	CACTTTGCCAAACACCACAT
hMAPT	GCGGGAAGGTGCAGATAAT	CTCCCAGGACGTGTTTGATATT
App	TCAGGGACCAAAACCTGCAT	GCACCAGTTCTGGATGGTCA
Clu	AGCGCACTGGAGCCAAG	TCAGAGACCTCCTGCTCTCC
Gapdh	TGGCAAAGTGGAGATTGTTGCC	AAGATGGTGATGGGCTTCCCG
Gfap	ACGACTATCGCCGCCAACT	GCCGCTCTAGGGACTCGTTC
Tm4sf1	AGCCAGGCCCAGGACTCAACT	GCCAGGCCCACAACGTACC
Cd14	GCCAGGCCCAGGACTCAAT	GCCAGGCCCACAACGTACC
Serpina3n	CAACCTTACAGGCCAACCCAT	GGGCACCAAGTAGTCCTAGATGCT
Stat3	CAACTTCAGACCCGCCAACA	GCCGCTCCACCACGAAGG
Serping	GTGAAGCTCTACCACGCCTT	CTGTCTCCAGCCCCAAGAAG
Vim	TCGAGGTGGAGCGGGACAAC	TGCAGGGTGCTTTCGGCTTC

In situ enzymatic activity

In situ enzymatic redox activity of major metabolic pathways was evaluated on coronal brain cryosections using a quantitative enzyme histo-biochemistry approach with the nitro blue tetrazolium (NBT) assay as described previously (Botman et al. 2014; Miller et al. 2017; Luczkowska et al. 2024). In this study, the enzymatic activities of glyceraldehyde-3-phosphate dehydrogenase (GAPDH), lactate dehydrogenase (LDH), succinate dehydrogenase (SDH), glutamate dehydrogenase (GDH), β-hydroxybutyrate dehydrogenase (BDH) and glucose-6-phosphate dehydrogenase (G6PDH) were measured using their respective enzyme-specific substrates and cofactors. Practically, cryopreserved brain tissue embedded in an optimal cutting temperature (OCT) compound was sectioned in a cryostat to yield 10 µm thick cryosections, which were then allowed to equilibrate at 37 °C in a dark, humid chamber before the reaction mix was added. The reaction mixture was prepared in a PBS buffer (0.1 M KH_2PO_4, 0.1 M Na_2HPO_4) supplemented with 18% polyvinyl alcohol (PVA, Alfa Aesar, Ward Hill, MA), a formulation that permits the free diffusion into the sectioned tissue of small molecules such as substrates and cofactors while keeping proteins localized at their in situ positions. At the of the incubation period determined by kinetic assays, the reaction was terminated by washing the slides with PBS at 60 °C for 10 minutes. The samples were then fixed in formalin solution 10% (Sigma-Aldrich), rinsed by Milli-Q water and mounted with glycerol based mounting medium.

Table: Composition of the NBT assay according to the targeted enzyme.

Enzyme	GAPDH	LDH	SDH	GDH	*BDH*	*G6PD*
pH of PBS	7.4	7.4	7.4	7.4	7.4	7.4
NBT (mM)	5	5	5	5	5	5
NAD^+ (mM)	3	1.5	0	3	3	0
ADP (mM)	0	0	0	2.0	0	0
NADP (mM)	0	0	0	0	0	5
$MgCl_2$ (mM)	0	0	0	0	0	1.5
NaN_3 (mM)	5	5	5	5	5	5
PMS (mM)	0.32	0.32	0.32	0.32	0.32	0.32
Substrate (mM) Supplier	A	B	C	D	E	F
Incubation (min)	45	30	30	60	30	15

A: glyceraldehyde-3-phosphate (1.5) Sigma-Aldrich; **B**: Lactate (2) Combi-Blocks, San Diego, CA; **C**: Succinate (0.7) Sigma-Aldrich, Burlington, MA; **D**: Glutamate (10) Sigma-Aldrich, Burlington, MA; **E**: D-3-hydroksubutyrate (16) Thermo Fisher Scientific; **F**: D-glucose-6-phosphate (10) Sigma-Aldrich

Supplemental figures

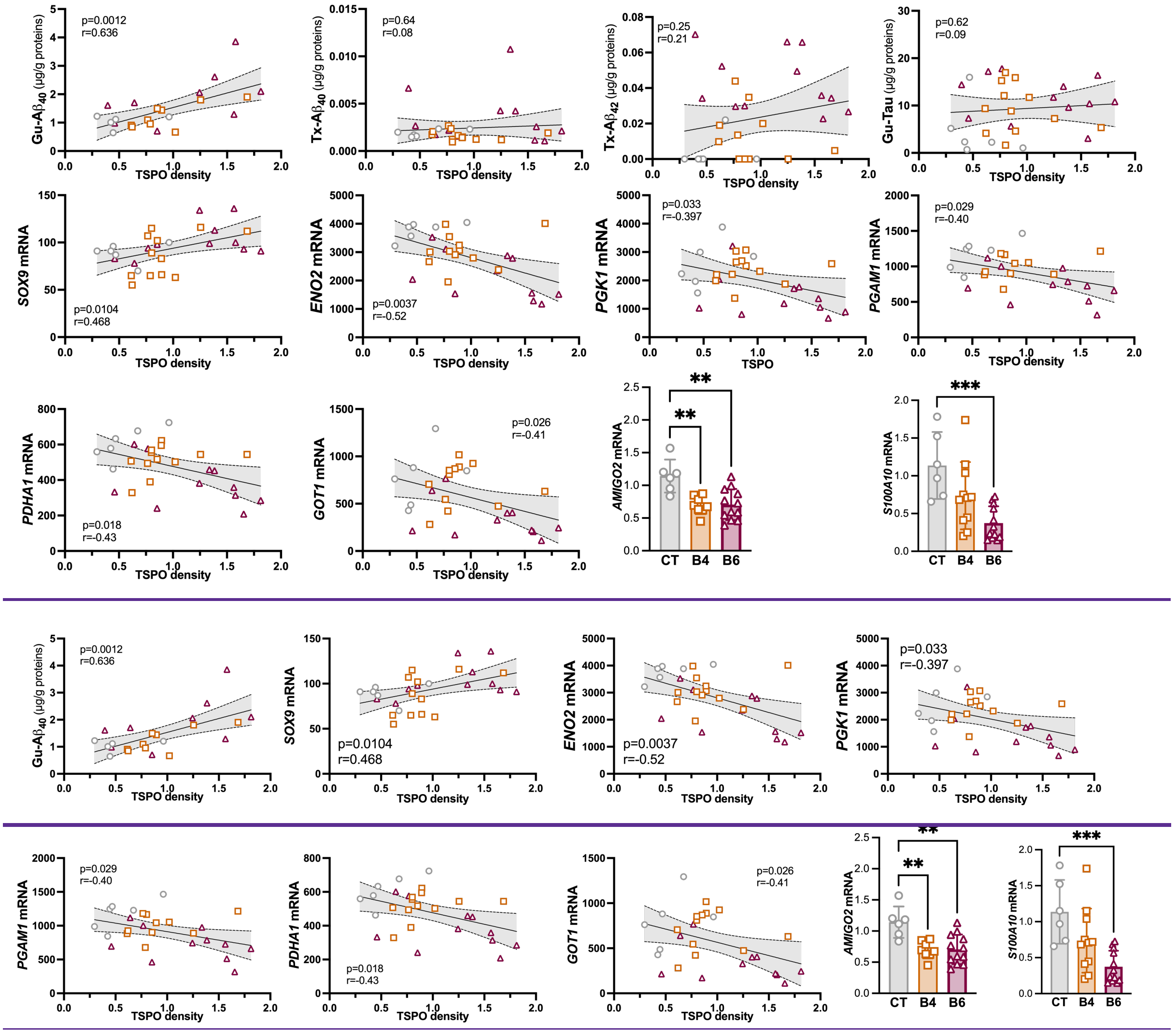

Supplemental Fig.1. TSPO density is correlated with amyloid, astrocytes and glycolysis markers. Correlation analysis (Pearson's coefficient) between TSPO (TSPO/ACTIN ratio) and Guanidine-soluble Aβ_{40} (Gu-Aβ_{40}, µg/g proteins, ELISA test) or mRNA expression levels of the specific *SOX9* astrocyte gene or genes involved in glycolysis (*ENO2, PGK1, PGAM1*, *PDHA1*, *GOT1*). *AMIGO2* and *S100A10* mRNA levels were determined by qPCR in control (CT, grey circles), Braak 4 (B4, orange squares) and Braak 6 (B6, purple triangles) subjects. Kruskal-Wallis test with the Dunn's multiple comparisons *post hoc* test.

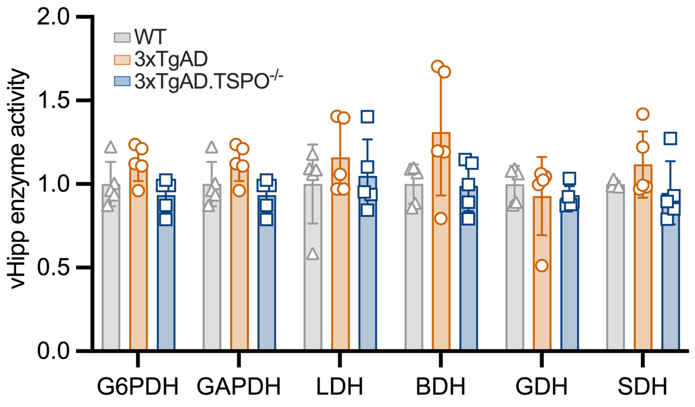

Supplemental Fig.2. Metabolic mapping in the ventral hippocampus.

Enzyme activity was measured in the ventral hippocampus (vHipp) of WT, 3xTgAD and 3xTgAD.TSPO$^{-/-}$ mice. Data are presented as individual values and mean ± SD (n=5/group) and analyzed by the Two-way ANOVA test with a LSD *post hoc* test (genotype, $F_{2,72}$=7.35, P=0.0012; enzyme, $F_{5,72}$=1.28, P>0.05; genotype x enzyme, $F_{10,72}$=0.75, P>0.05; LSD posthoc test for genotype: 3xTgAD vs WT, P=0.006; 3xTgAD vs 3xTgAD.TSPO$^{-/-}$, P=0.0005; 3xTgAD.TSPO$^{-/-}$ vs WT, P>0.05).

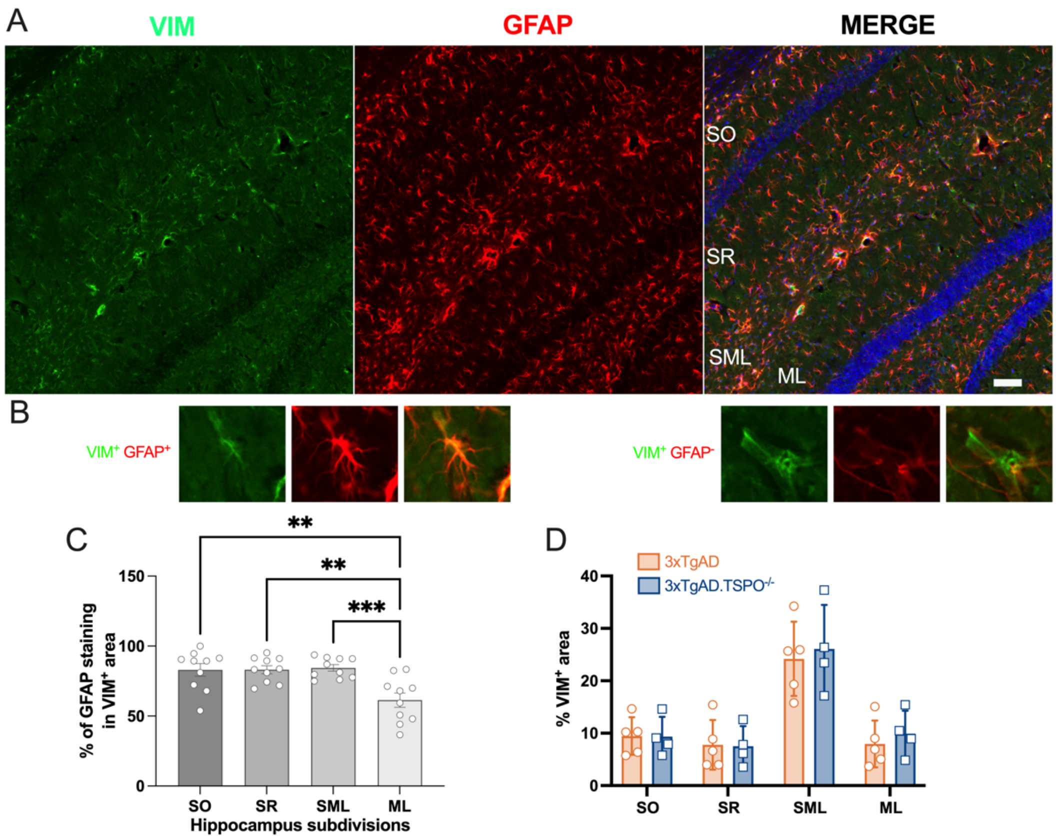

Supplemental Fig.3. VIMENTIN⁺ area is mainly colocalized with GFAP⁺ area.

A, Colocalization between VIMENTIN (VIM, green) and GFAP (red) in the subdivisions of the hippocampus: *stratum oriens* (SO), *stratum radiatum* (SR), *stratum lacunosum-moleculare* (SML) and molecular layer of dentate gyrus (ML). **B,** Example of a VIM^+GFAP^+ and VIM^+GFAP^- cells from images showed in A. **C,** Quantification of % $GFAP^+$ area in VIM^+ area demonstrated high level of colocalization except in ML (one-way ANOVA: subdivision effect $F_{3,36}$=8.23, P<0.001, post hoc Tukey's test: ML vs SO and SR, *P<0.01*; ML vs SML, *P<0.001*). **D,** Quantification of % of positive VIM-ir area in the subdivisions of the dorsal hippocampus (two-way ANOVA: genotype x subdivision, $F_{3,28}$=0.11, P>0.05; genotype, $F_{1,28}$=0.24, P>0.05; subdivision, $F_{3,28}$=21.85, P<0.001).

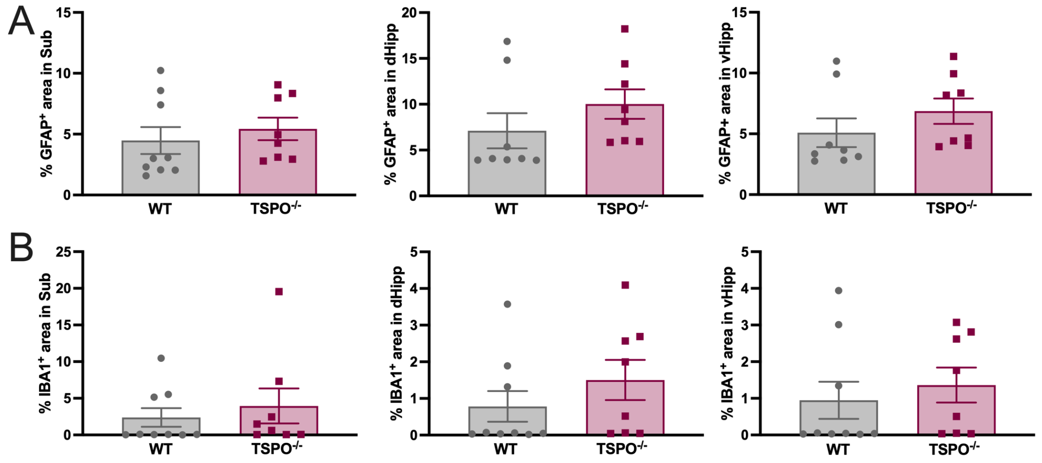

Supplemental Fig.4. TSPO$^{-/-}$ mice did not show glial activity changes.

Immunofluorescence analysis of astrocytes and microglia in 9-month-old WT and TSPO$^{-/-}$ mice**. A,** Quantification of % of positive GFAP-ir area in the subiculum (Sub), dorsal hippocampus (dHipp) and ventral hippocampus (vHipp) (two-tailed unpaired t-test: P>0.05). **B,** Quantification of % of positive IBA1-ir area in Sub, dHipp and vHipp (two-tailed unpaired t-test: P>0.05).

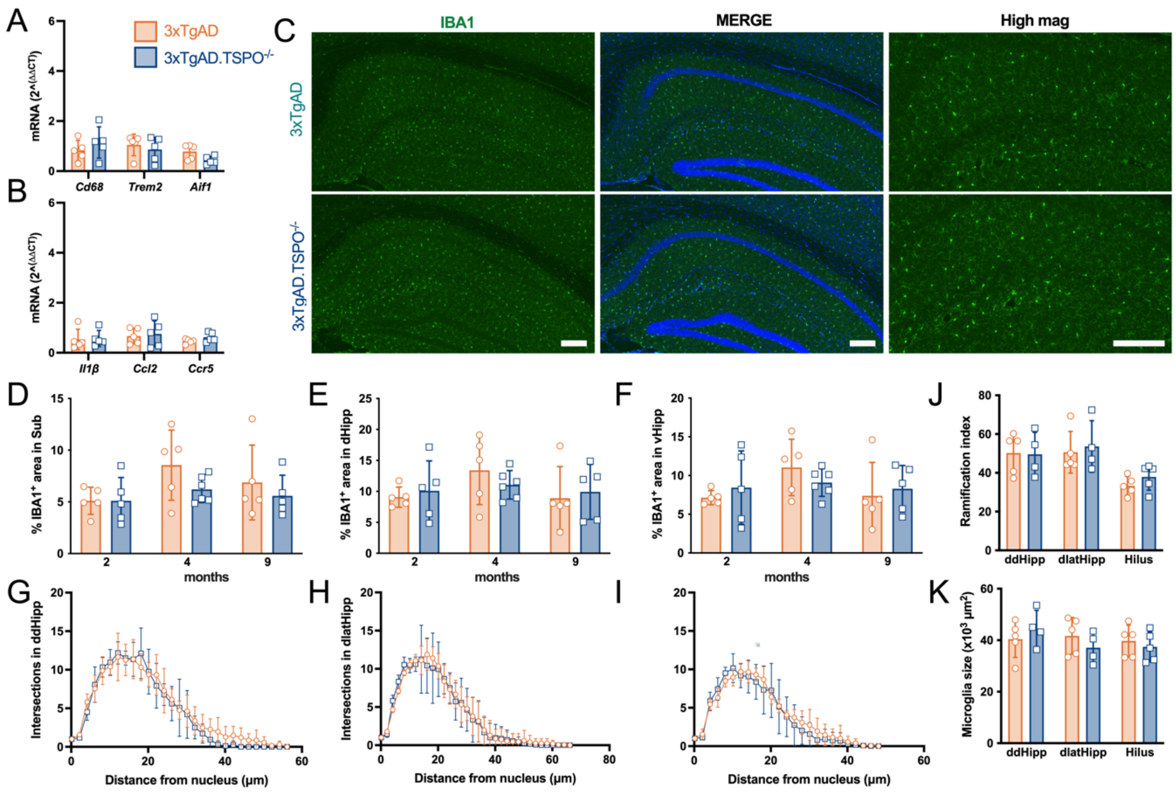

Supplemental Fig.5. 3xTgAD.TSPO$^{-/-}$ mice did not show microglial marker changes. **(A)** Quantification of mRNA (2^-$^{(\Delta\Delta CT)}$) levels in the whole hippocampus of microglial genes (two-way ANOVA, genotype, $F_{1,24}$=0.20, P>0.05; gene, $F_{2,24}$=2.37, P>0.05; genotype x gene, $F_{2,24}$=1.69, P>0.05). **(B)** Quantification of mRNA (2^-$^{(\Delta\Delta CT)}$) levels in the whole hippocampus of inflammatory genes (two-way ANOVA, genotype, $F_{1,24}$=0.57, P>0.05; gene, $F_{2,24}$=0.78, P>0.05; genotype x gene, $F_{2,24}$=0.08, P>0.05). (**C**) Representative example of DAPI (blue) and IBA1 (green) immunoreactivity at two magnifications in 9-month-old 3xTgAD and 3xTgAD.TSPO$^{-/-}$ mice. Scale bars: 200 μm. **(D-F)** Quantification of % of positive IBA1-ir area in the subiculum (Sub, D), dorsal hippocampus (dHipp, E) and ventral hippocampus (vHipp, F) (%IBA1^{+}, two-way ANOVA, Subiculum: genotype x age, $F_{2,25}$=0.60, P>0.05; genotype, $F_{1,25}$=1.89, P>0.05; age, $F_{2,25}$=2.26, P>0.05; dHipp: genotype x age, $F_{2,25}$=0.57, P>0.05; genotype, $F_{1,25}$=0.004, P>0.05; age, $F_{2,25}$=1.51, P>0.05; vHipp: genotype x age, $F_{2,25}$=0.76, P>0.05; genotype, $F_{1,25}$=0.005, P>0.05; age, $F_{2,25}$=1.65, P>0.05). **(G-I)** Quantification by Sholl analysis of the number of intersections as function of the distance from soma in dorso-dorsal (ddHipp, G), dorso-lateral (dlatHipp, H) hippocampus and hilus (I) (two-way ANOVA, ddHipp: genotype x distance, $F_{28,196}$=0.64, P>0.05; genotype, $F_{1,7}$=0.44, P>0.05; distance, $F_{28,196}$=78.9, P<0.0001; dlatHipp: genotype x distance, $F_{33,231}$=0.37, P>0.05; genotype, $F_{1,7}$=0.02, P>0.05; distance, $F_{33,231}$=86.8, P<0.0001; hilus: genotype x distance, $F_{24,192}$=0.74, P>0.05; genotype, $F_{1,8}$=0.24, P>0.63; distance, $F_{24,192}$=72.9, P<0.0001). **(J)** Total number of ramifications (two-way ANOVA: genotype x area, $F_{2,22}$=0.18, P>0.05; genotype, $F_{1,22}$=0.39, P>0.05; area, $F_{2,22}$=8.06, P=0.0024). **(K)** Size of the soma of microglia (two-way ANOVA: genotype x area, $F_{2,22}$=1.06, P>0.05; genotype, $F_{1,22}$=0.14, P>0.05; area, $F_{2,22}$=0.91, P>0.05).

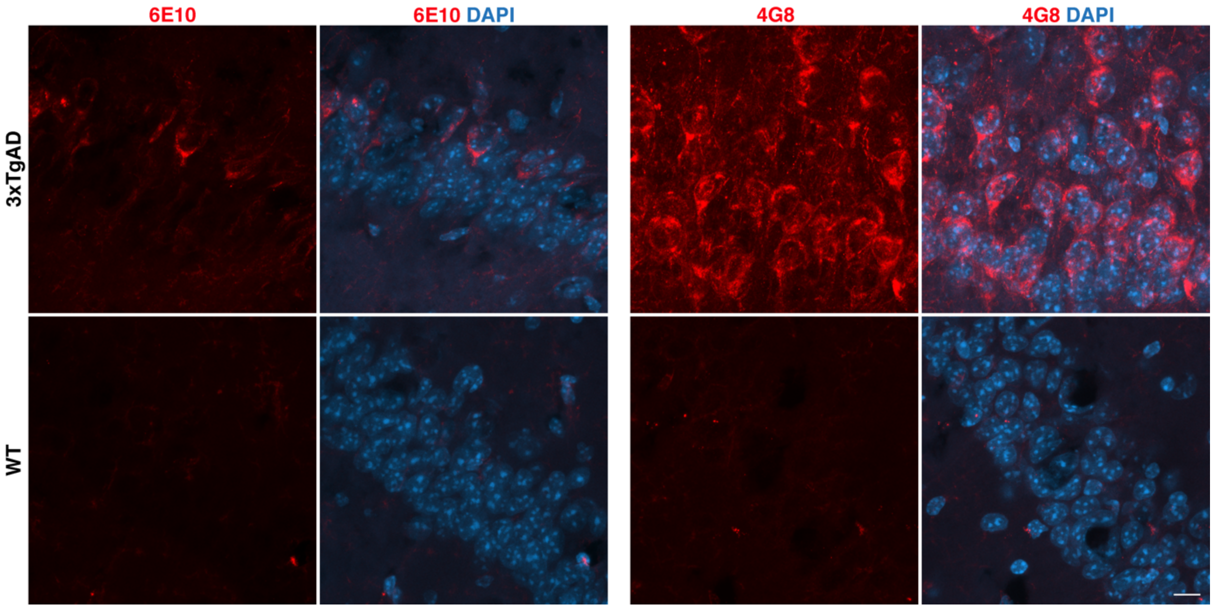

Supplemental Fig.6. 6E10 and 4G8 immunoreactivities are absent in wild-type mice.

Representative example of 6E10 or 4G8 perinuclear immunoreactivity (red) and DAPI (blue) in 9-month-old 3xTgAD and WT mice. WT mice are negative for both staining. Scale bar: 10 µm.

Supplemental Tables

	Sex	Age	Postmortem delay (h)	Braak level
Netherland brain bank				
Non-demented control	2 M / 4 F	90 ± 7.1 (82-101)	6.65 ± 1.7 (4.3-9.5)	1 B2; 5 B3
AD Braak 4	4 M / 8 F	85.9 ± 7.8 (76-102)	4.89 ± 1.9 (3.0-10)	12 B4 ***
AD Braak 6	4 M / 9 F	82.8 ± 5.9 (75-95)	5.4 ± 1.5 (2.8-8.0)	1 B5, 12 B6 ***,§§§
Geneva Brain Bank (TSPO in Astrocytes)				
Non-demented control	2 M / 2 F	86 ± 10.8 (71-96)	31 ± 6.2 (26-40)	2 B2; 2 B3
AD Braak 6	3 M / 1 F	86.7 ± 6.7 (77-92)	25.2 ± 7.8 (19-36)	2 B5; 2 B6*
Geneva Brain Bank (TSPO in Astrocytes and Microglia)				
Non-demented control	3 M / 3 F	79.2 ± 5.3 (73-86)	43.5 ± 21.5 (28-84)	1 B0; 3 B2; 2 B3

Supplemental Table. 1 Characteristics of human samples.

Netherland brain bank. The three groups did not differ neither in sex distribution (X^2=0.02, P=0.98), postmortem delay (one-way ANOVA: $F_{2,28}$=2.08, P=0.14), nor in age (one-way ANOVA: $F_{2,28}$=2.24, P=0.12). In contrast, Braak level was different between groups (one-way ANOVA: $F_{2,28}$=365, P<0.0001, post hoc Holm-Sidak test: AD Braak 4 vs non-demented, ***P<0.0001; AD Braak 6 vs non-demented, ***P<0.0001; AD Braak 6 vs AD Braak 4, §§§P<0.0001). ***Geneva brain bank*** (***TSPO in Astrocytes)***. The two groups did not differ neither in sex distribution (X^2=0.53, P=0.46), postmortem delay (Mann-Whitney: U=3.5, P=0.25), nor in age (Mann-Whitney: U=8, P>0.99). In contrast, Braak level was different between groups (Mann-Whitney: U=0, P=0.028). Age and postmortem delay are presented as mean ± SD and the range. B: braak level; F: females; M: males.
